# Molecular and biological investigating of tea plant necrotic ring blotch virus as a worldwide threat

**DOI:** 10.1038/s41598-023-46654-3

**Published:** 2023-11-04

**Authors:** Fereshteh Esmaeilzadeh, Abozar Ghorbani, Davoud Koolivand

**Affiliations:** 1 Department of Plant Protection, Faculty of Agriculture, University of Zanjan, Zanjan, 45371-38111 Iran; 2 Nuclear Agriculture Research School, Nuclear Science and Technology Research Institute (NSTRI), Karaj, Iran

**Keywords:** Biological techniques, Evolution, Genetics, Molecular biology

## Abstract

*Tea plant necrotic ring blotch virus* (TPNRBV) has emerged as a significant threat to tea plantations, primarily in China. Since 2020, similar symptoms have been observed in tea plants in northern Iran, raising concerns about the spread of this viral infection. In this study, we conducted an extensive investigation involving approximately 70 samples collected from both symptomatic and asymptomatic tea plants. Using reverse transcription-polymerase chain reaction with specially designed primers, we successfully amplified DNA fragments from 26 samples, confirming the presence of TPNRBV. Subsequent sequencing of these fragments revealed various segments of the TPNRBV genome. Our phylogenetic analysis revealed that the Iranian TPNRBV isolates formed a distinct sub-cluster alongside Chinese isolates, distinguishing them from Japanese isolates. These finding sheds light on the genetic diversity and relationships of TPNRBV across different regions. Additionally, we explored the potential modes of TPNRBV transmission. Mechanical transmission experiments confirmed the ability of the virus to infect *Nicotiana rustica* and *Chenopodium quinoa* seedlings, highlighting the risk of mechanical spread within tea plantations. Moreover, we investigated seed transmission and found evidence of TPNRBV in various parts of tea seeds, suggesting the possibility of seed-borne transmission. Overall, this comprehensive study enhances our understanding of the biological and molecular characteristics of TPNRBV, an emerging threat to global tea production. Our findings provide valuable insights into the virus’s transmission dynamics and genetic diversity, which are essential for developing effective management strategies to mitigate its impact on tea cultivation worldwide.

## Introduction

Tea (*Camellia sinensis* (L.) O. Kuntze) is a perennial evergreen plant from Theaceae, highly valued for its leaves that are processed to produce one of the world’s most popular non-alcoholic beverages. In addition to its popularity as a beverage, tea contains various secondary metabolites, such as catechins, theanine, and caffeine, known for their potential health^1–3^ benefits (1–3). The global tea industry plays a pivotal role in the agricultural economy, contributing significantly to the income of many developing countries. China stands as the world’s leading tea producer, with India, Kenya, and other nations also making substantial contributions to global tea production. Iran, although not among the top tea-producing nations, maintains a tea cultivation industry covering approximately 15,000 hectares and an annual production of around 85,000 tons (FAO 2021).

However, the tea cultivation industry faces several challenges, including biotic stressors such as fungi, nematodes, and bacterial pathogens, which can lead to substantial yield losses^4–7^. Recent advances in metagenomic sequencing have unveiled the presence of several plant viruses in tea plants. Among these viruses, tea plant necrotic ring blotch virus (TPNRBV) and tea plant line pattern virus (TPLPV) have emerged as some of the most prevalent^8^. TPNRBV, in particular, has been identified as a significant threat to tea plantations, primarily in China, and has been detected in Japan and Iran as well^9,10^.

TPNRBV belongs to the genus Blunervirus in the family Kitaviridae and possesses a segmented genome (RNA1, RNA2, RNA3, and RNA4)^11^. It primarily affects tea plants, leading to symptoms such as necrotic ring blotches and other leaf abnormalities. While the mode of TPNRBV transmission has not been fully elucidated, it has been suggested that insect vectors, such as mites, aphids, leafhoppers, and whiteflies, may play a role in its spread^12^. These infections not only compromise the health of tea plants but also impact tea production, as the leaves are the primary source of tea products.

Despite the potential economic impact of TPNRBV on tea production, there is limited information available regarding its epidemiology, population genetic structure, transmission modes, host range, and effective control methods. This lack of knowledge poses challenges for developing and implementing management strategies. In Iran, where TPNRBV has been detected, a comprehensive study on the virus’s prevalence and genetic diversity is lacking, particularly in the central tea-growing regions of the country.

The present study aims to address these knowledge gaps by investigating the infection status of TPNRBV in tea plantations in northern Iran. We also explore the distribution, mode of transmission and genetic diversity of TPNRBV in Iranian tea plantations. This research provides valuable insights into the biology and epidemiology of TPNRBV, which are essential for developing effective management strategies to mitigate its impact on tea cultivation globally.

## Results

### Field observations

**Figure 1:**
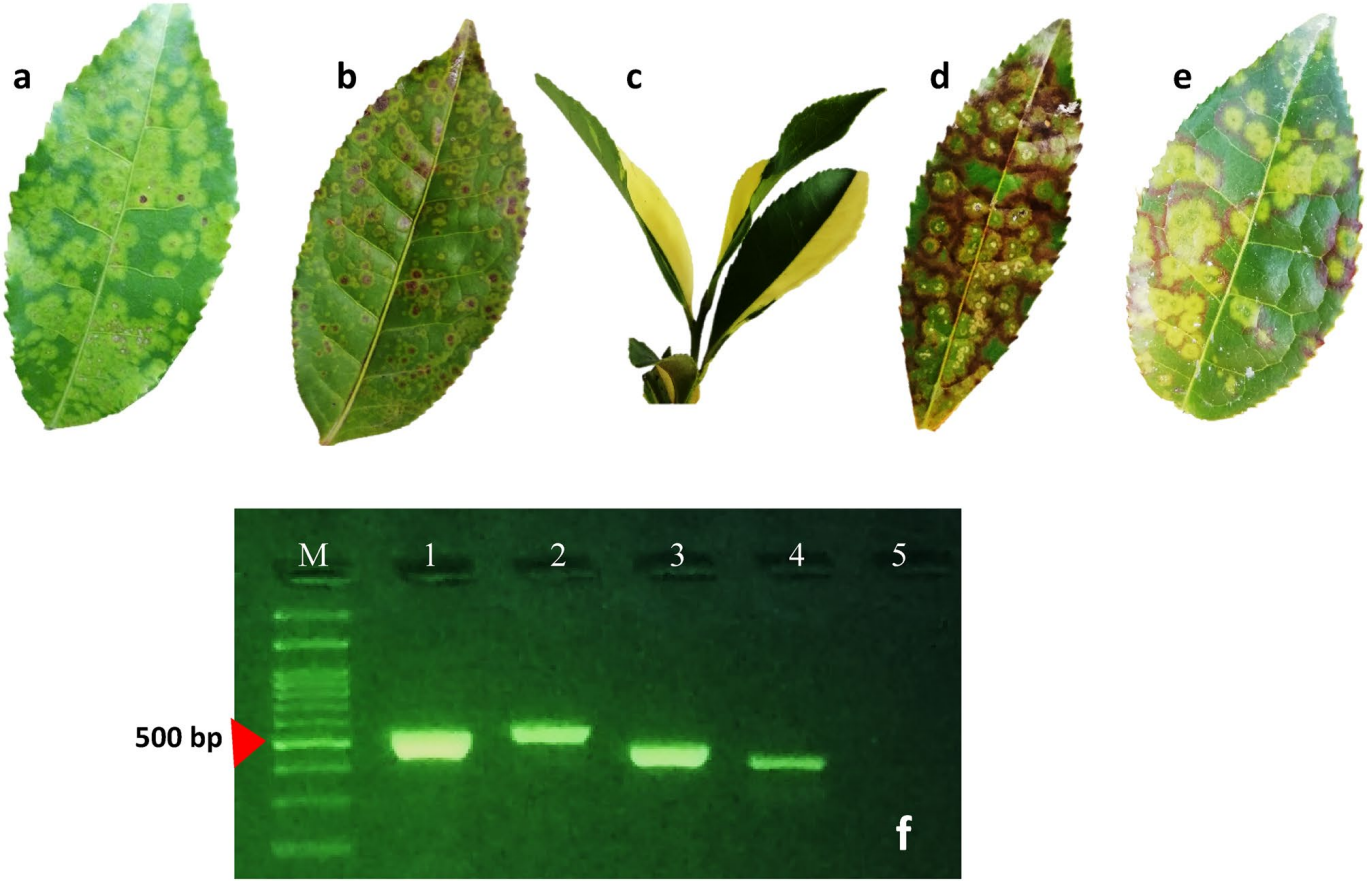
Symptoms caused by the tea plant necrotic ring blotch virus (TPNRBV) in tea plants: (**a**,**b**) necrotic ring blotch (**c**) chlorina. (**d**) brown rings. (**e**) Red–brown rings. (**f**), electrophoresis of PCR product amplified from RNA1, RNA2, RNA3, and RNA4 a positive sample by TPNRBV specific primers.

A wide array of symptoms, including necrotic ring blotch, chlorina, reddish-brown rings, brown rings, and yellowing, were consistently observed in the most frequently visited tea plantations Fig. 1.

The hallmark symptom of TPNRBV infection, necrotic ring blotches on mature leaves, was consistently present across all surveyed tea plantations. In one of the plantations, reddish-brown and brown rings were prevalent on the leaves, in contrast to the more common symptoms observed elsewhere. These symptoms typically manifested from early autumn to late winter and gradually subsided with rising temperatures. While chlorina leaves were occasionally observed in some plantations, ring spots were notably found on the pods of severely infected shrubs in several tea orchards. Interestingly, there were no observable symptoms on the flowers or green shoots.

### TPNRBV presence in symptomatic plants

**Figure 2:**
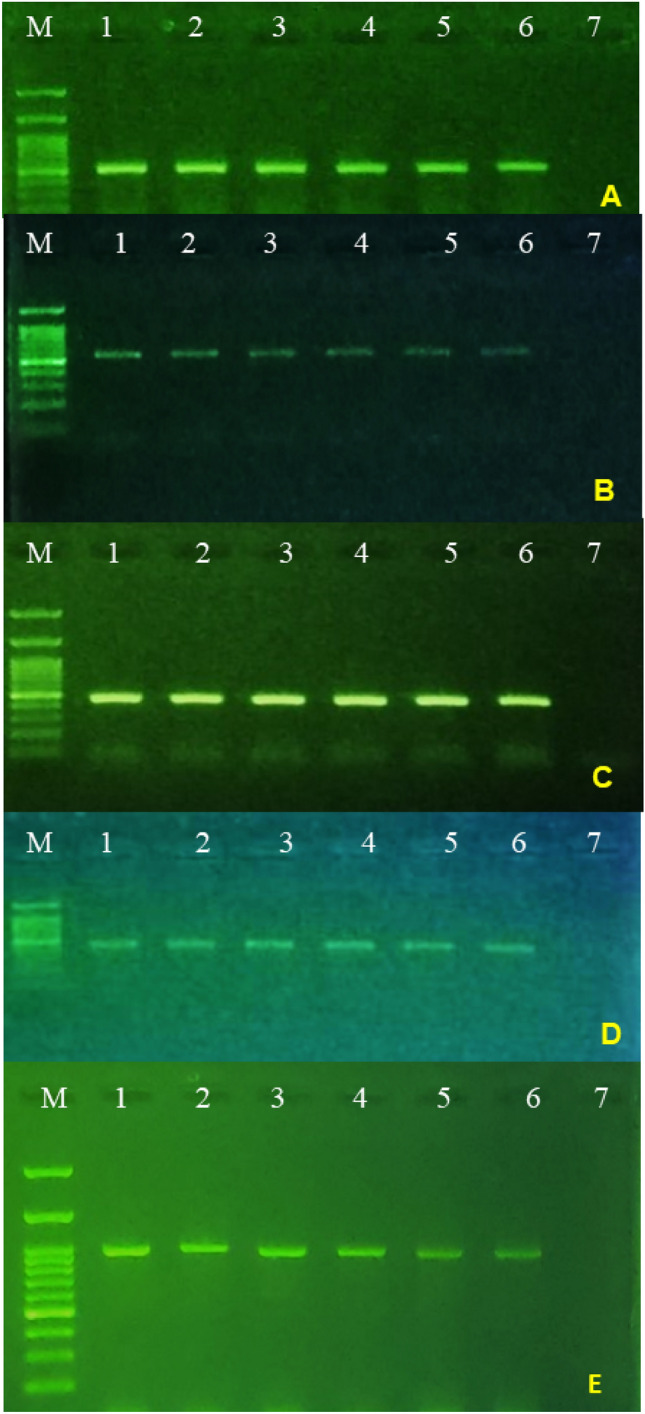
Detection of TPNRBV in infected tea plants using reverse transcription polymerase chain reaction: Agarose gel electrophoresis analysis of different segments of TPNRBV and MP gene amplified from infected tea plants by primer pairs corresponding to a part of RNA1, RNA2, RNA3, RNA4, and MP of TPNRBV; M:1.5 kb Marker; 1: RNA1; 2: RNA2; 3: RNA3; 4: RNA4; Movement Protein (MP) gene.

Among the 26 symptomatic samples, reverse transcription-polymerase chain reaction (RT-PCR) with TPNRBV-specific primers successfully amplified the expected DNA fragments (535 bp, 551 bp, 459 bp, 407 bp, and 948 bp related to RNA1, RNA2, RNA3, RNA4, and MP genes, respectively) Fig. 2. Subsequent BLAST analysis of the sequenced amplicons confirmed their correspondence to specific segments of the TPNRBV genome. This result established that approximately half of the collected plants displayed symptoms due to TPNRBV infection, suggesting a relatively high prevalence of the virus in Iranian tea orchards. Notably, mixed infections involving TPLPV, BNRBV, or APLPV were not detected in TPNRBV-positive samples. This indicates that co-infections with other viruses are likely infrequent, at least among the examined viruses. We identified a total of 11 TPNRBV isolates, with their nucleotide sequences deposited in GenBank under the accession numbers listed in Table 2. Comparative analysis of the Iranian isolates revealed nucleotide identities ranging from 92 to 98% similarity with TPNRBV sequences available in GenBank (NC_040401-NC_040404 and MG781152-MG781155).

### Phylogenetic analysis

**Figure 3:**
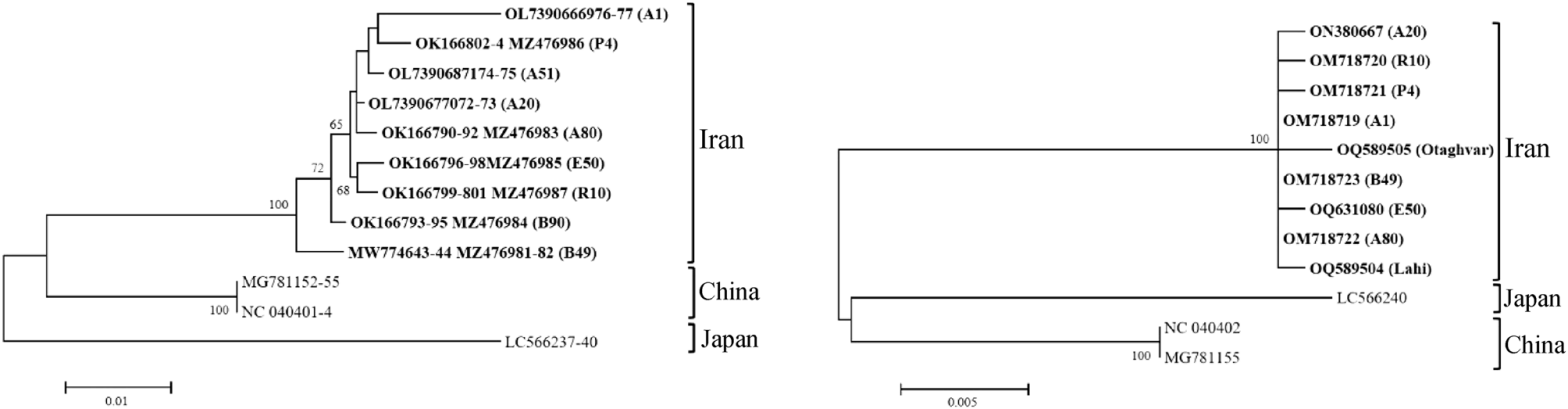
Phylogenetic trees constructed with sequences of new isolates and related sequences of TPNRBV using the Maximum Likelihood method in MEGA11. Left) based on the consensus sequences from RNA1, RNA2, RNA3, and RNA4.; Right) based on the MP gene sequences. Bootstrap analysis was applied using 1000 replicates. Bootstrap values (1000 replications) below 50% are not shown.

Phylogenetic analysis based on RNA segments demonstrated that the Iranian TPNRBV isolates clustered in distinct groups similar to isolates from China and Japan, with these relationships further supported by phylogenetic analysis based on MP (movement protein) sequences Fig. 3. Specifically, the RNA segment-based phylogenetic tree indicated that Iranian isolates formed a primary cluster with two reported isolates from China, while the Japanese isolate was distinct from this primary group. These results suggest that Iranian isolates share a closer phylogenetic affinity with Chinese isolates, implying a common ancestry. Overall, the analysis revealed three distinct subclusters, encompassing Iranian, Chinese, and Japanese isolates Fig. 3, based on both MP and RNA segment sequences. Notably, Iranian isolates occupied an independent subcluster in the MP-based phylogenetic tree, suggesting some genetic divergence between Iranian, Chinese, and Japanese isolates Fig. 3. However, due to the limited availability of global TPNRBV sequences, conclusive origins of the virus remain challenging to ascertain.

### Mechanical inoculation with TPNRBV

**Figure 4:**
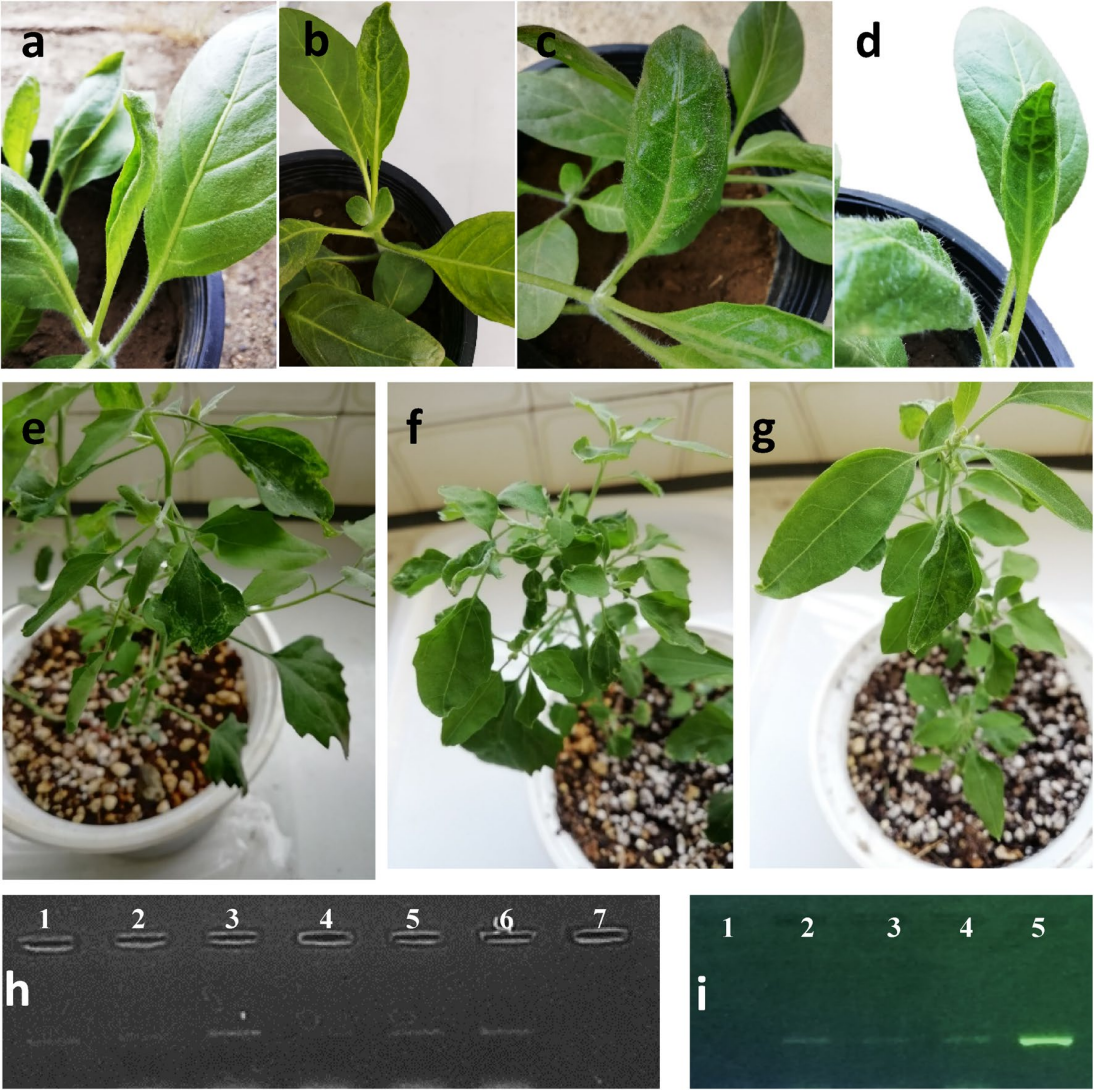
Systemic symptoms caused by TPNRBV R10 and A1 isolates in inoculated *N. rustica* and *C. quinoa* plants: (**a**) leaf deformation (upward curling of leaflet margin), (**b**) leaf and vein curling, (**c**) vein/veinal swelling, and (**d**) extreme leaf distortion, (**e**) Ring Pattern symptoms on leaves; (**f**) extreme distortion of leaves; (**g**) mosaic of leaves. (**h**) PCR product electrophoresis from inoculated *N. rustica* by TPNRBV primer, 1–6: different inoculated plants, 7: negative control (**i**) PCR product electrophoresis from inoculated *C. quinoa* by TPNRBV primer, 1: negative control, 2–4: different inoculated plants, 5: Positive control.

Mechanical inoculation of *Nicotiana rustica* and *Chenopodium quinoa* seedlings with TPNRBV isolates R10 and A1 resulted in the development of various symptoms. These symptoms included leaf deformation, leaf and vein curling, vein/veinal swelling, bright yellow mosaic of leaves, extreme leaf distortion, and severe upward leaf curling symptoms, with observations made at 30–35 days post-inoculation (dpi) Fig. 4. Subsequent RT-PCR analysis confirmed the presence of TPNRBV in mechanically inoculated *N. rustica* and *C. quinoa* plants Fig. 4, verifying the success of mechanical transmission. In contrast, control plants inoculated with a buffer solution displayed no symptoms or amplification. These findings underscore the ability of TPNRBV to be mechanically transmitted, contrary to previous reports, and emphasize the potential for short-distance virus transmission within tea plantations. Additionally, this mode of transmission suggests that tools and equipment used in tea plantations, such as leaf-cutting scissors and harvester machines, can inadvertently contribute to virus spread. Implementing effective disinfection procedures for these tools is crucial for preventing viral dissemination.

### Seed transmission of TPNRBV

**Figure 5:**
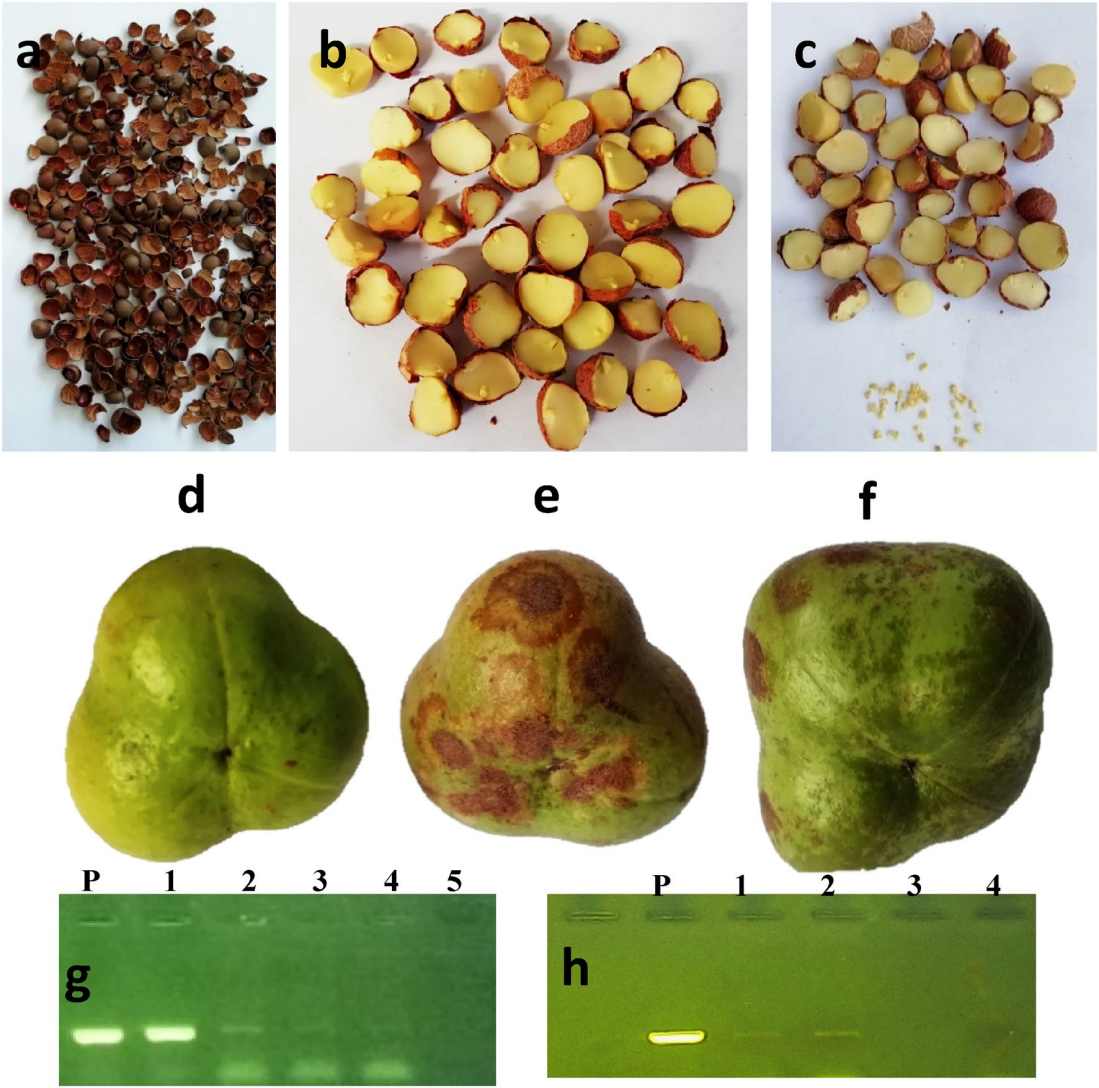
The result of seed-borne and seed-transmitted nature of TPNRBV in *C. sinensis.* (**a**) seed coat, (**b**) cotyledon, (**c**) embryo, (**d**) seed pod from asymptomatic plant, (**e**,**f**) TPNRBV symptoms in infected pods, (**g**) PCR-Product electrophoresis from different parts of seed by TPNRBV primer, P: Positive control, 1: Pod, 2: embryo, 3: cotyledon, 4: seed coat, 5: negative control, (**h**) PCR-Product electrophoresis from seedling that grown from infected seeds by TPNRBV primer, P: Positive sample, 1 and 2: PCR products amplified from seedlings, two seedlings as representative, 3: healthy plant, 4: negative control for PCR.

Our investigation of TPNRBV seed transmission revealed that 26 out of 40 planted seeds, sourced from TPNRBV-infected plants, germinated from fallen seeds collected in various tea plantations. Among these seedlings, three displayed symptoms consistent with necrotic ring blotch infection after approximately 6 months, while the remaining seedlings remained asymptomatic. Notably, TPNRBV was detected in three symptomatic seedlings and two randomly selected asymptomatic ones. Furthermore, RT-PCR results confirmed the presence of TPNRBV in different parts of the collected seeds from infected plants, highlighting the ability of the virus to infect various seed structures. The faint bands observed in the gel electrophoresis of seed coat, cotyledon, and embryo extracts indicated a low concentration of TPNRBV in infected seeds. Conversely, TPNRBV was not detected in seeds collected from asymptomatic plants. Additionally, symptomatic pods derived from infected plants and seedlings were confirmed to be infected by TPNRBV through RT-PCR analysis Fig. 5. This comprehensive assessment underscores the seed-borne and seed-transmitted nature of TPNRBV in tea plantations. While not all seedlings from infected seeds were infected, indicating less than 100% transmission, the possibility of uneven virus distribution in the seedlings could not be ruled out. Seed transmission of plant viruses can be influenced by various factors, including virus strain, host species/genotype, and environmental conditions. Therefore, further research is needed to determine the extent and implications of TPNRBV seed transmission.

## Discussion

In this study, we provide the first comprehensive description of the tea-infecting TPNRBV population in Iran, encompassing an analysis of induced symptoms, genetic characterizations of distinct virus isolate within this population, and the modes of TPNRBV transmission.

### Symptomatology and factors influencing variation

The TPNRBV-infected tea plants exhibited a spectrum of symptoms, the diversity of which may be attributed to various factors. Genetic factors, such as the tea cultivar and genetic variation of the virus, likely play a role in shaping symptom variation^8,13^. Environmental factors are also influential, especially in the context of tea cultivation practices in Iran. Unlike some regions where tea plants are propagated from selected adult plants, Iranian tea plantations predominantly consist of seed-grown individual shrubs. These variations in growth conditions result in morphological, physiological, and functional disparities among shrubs, which could contribute to the observed range of symptoms.

Another noteworthy factor impacting symptomatology is the presence of co-infecting viruses. While mixed infections of TPNRBV and TPLPV have been reported in Chinese tea plantations^8^, our study did not detect such co-infections. Several factors could account for this disparity, including differences in tea cultivars, the possibility of TPNRBV inducing a priority effect that inhibits other viral infections^13^, or variations in the prevalence of different viruses in Iranian tea plants. To address these questions, a comprehensive characterization of the Iranian TPNRBV population is warranted, which, aside from a brief report on TPNRBV from a limited area in Mazandaran based on RNA2, remains largely unexplored^13^. Conducting virome analyses of tea plants can facilitate the identification of associated viruses, elucidate mixed infections, and contribute to the current dearth of scientific literature on tea viruses in Iran.

### Genetic diversity and phylogenetic relationships

In addition to virus-induced symptoms, we delved into the genetic diversity of multiple TPNRBV isolates. The availability of TPNRBV sequences in public databases is notably limited, with most sequences originating from China and Japan. Our study represents the largest dataset to date, albeit still limited, making it challenging to draw definitive conclusions regarding the genetic diversity and evolution of TPNRBV^8^. Nevertheless, our phylogenetic analyses suggest a relatively close genetic relationship between Iranian TPNRBV isolates and those from China, indicating a shared common ancestor.

Furthermore, the genetic structure of TPNRBV populations based on geographical origin suggests ongoing processes of virus adaptation and possible genetic isolation among populations in^13^ different countries^14^. However, to substantiate these observations, a more extensive exploration of the global genetic diversity of the TPNRBV population is essential.

### Transmission dynamics

Our investigation into TPNRBV transmission modes yielded significant insights. In contrast to prior studies^8,12^, we demonstrated that mechanical transmission of TPNRBV is feasible^8,12^. Several variables can influence mechanical transmission, including the virus isolate, plant species used as indicators, geographical and environmental conditions, and inoculation techniques. These factors may account for discrepancies between our findings and previous results. Moreover, the timing of virus presence analysis, such as the detection at 75 days post-inoculation in our study versus 36 days in an earlier study, could also explain the variations. Notably, symptoms in shrubs are often observed relatively late after infection, suggesting low virus^14^ titers in the early stages post-inoculation.

Mechanical transmission of TPNRBV carries substantial implications for the tea industry. Tools such as tea leaf-cutting scissors, harvester machines, and pruning tools can potentially facilitate the short-distance transmission of the virus. Given the prevalent use of tea leaf-cutting scissors in Iranian plantations, this mode of transmission presents challenges, even with low infection levels. Investigating the efficacy of disinfection procedures and emphasizing sanitation and the use of healthy cuttings for new plantations are critical steps in mitigating viral spread. Additionally, while no insect vectors have been identified for TPNRBV transmission, considering the potential role of insects is prudent, especially given suggestions of eriophyid mites as vectors for related *Blunervirus* species^15–19^.

### Seed transmission

Historically, tea plants were propagated exclusively from seeds until the mid-nineteenth century, resulting in significant genetic diversity due to heterozygosity among seed-derived cultivars^20,21^. In contemporary tea cultivation in Iran and many other tea-producing regions, cuttings are commonly used for propagation, with seeds reserved primarily for breeding and cultivar development. Our study, however, provides evidence that seed transmission of TPNRBV is possible, with infected seeds leading to symptomatic seedlings and the detection of the virus in various seed structures.

Our findings suggest that the rate of TPNRBV seed transmission from infected seeds to seedlings is not 100%, aligning with observations made for other plant viruses (23). The presence of TPNRBV in all parts of the seed, including the seed coat, cotyledon, and embryo, indicates that the virus is both seed-borne and seed-transmitted in tea plantations. Nevertheless, the low virus concentration in infected seeds may contribute to the challenge of detecting the virus in seedlings. To address these nuances, future research should explore the mechanisms governing seed transmission.

## Conclusion

In this comprehensive study, we have conducted a thorough examination of the emergence and potential threat posed by the Tea Plant Necrotic Ring Blotch Virus (TPNRBV) to tea plantations, with a specific focus on northern Iran. Our research has provided critical insights into the biological and molecular attributes of TPNRBV and their implications for global tea production.

Our findings underscore TPNRBV as a substantial threat to tea plantations, with consistent symptomatology observed, including necrotic ring blotches, chlorina, reddish-brown rings, and yellowing, across affected tea plants. These symptoms were widespread in the surveyed tea plantations, underscoring the urgency of understanding and managing this viral infection. Moreover, our genetic characterizations have unveiled close genetic ties between Iranian TPNRBV isolates and those from China, suggesting a shared ancestry.

Additionally, we have demonstrated the mechanical transmission of TPNRBV, highlighting its significance in the context of disease management within the tea industry. The potential role of seed transmission adds another layer of complexity to the spread of TPNRBV, necessitating further research.

In summary, our study contributes substantially to our comprehension of the emerging threat posed by TPNRBV to global tea production. By shedding light on transmission dynamics, genetic diversity, and symptomatology, we pave the way for the development of effective management strategies for safeguarding tea cultivation worldwide. Our study emphasizes the importance of breeding programs targeting resistant cultivars and proactive disease control measures to mitigate the impact of TPNRBV on the tea industry.

## Limitations of the study

While our study yields valuable insights, certain limitations must be acknowledged:

1. *Sampling Bias* Our reliance on observations of visibly infected tea plants in northern Iran may introduce sampling bias, potentially underrepresenting asymptomatic or mildly symptomatic infections.
2. *Environmental Variability* We did not extensively investigate environmental factors that may influence virus transmission and symptom expression, a factor worth considering in future research.
3. *Limited Virus Sequences* The scarcity of TPNRBV sequences in public databases limits the depth of our phylogenetic analyses. Expanding sequence databases is essential for more comprehensive evolutionary studies.
4. *Seed Transmission Rate* Our study demonstrates seed transmission of TPNRBV, but the transmission rate from infected seeds to seedlings is not 100%. The mechanisms governing this variable transmission rate warrant further investigation.
5. *Co-infections and Vector Transmission* We did not explore the potential presence of co-infections with other viruses in tea plants, which could interact and influence TPNRBV symptoms and prevalence. Additionally, the role of insect vectors in TPNRBV transmission remains largely unexplored and warrants further investigation.

In conclusion, while this study significantly contributes to our knowledge of TPNRBV, these limitations remind us of the complexity of viral infections in agricultural systems. Future research should address these limitations to provide a more comprehensive understanding of TPNRBV and its impact on tea plantations.

## Material and methods

### Sample collection

Between 2020 and 2022, we collected samples of tea plant leaves, shoots, and seeds from various tea plantations in northern Iran. These samples exhibited symptoms such as necrotic ring blotch, chlorina, albino, brown rings, reddish-brown discoloration, as well as asymptomatic ones. Northern Iran plays a significant role in global tea production. Upon collection, the samples were promptly transported to the laboratory in ice bags and stored at − 80 °C until further processing. Our research involving the collection of tea plant samples adhered to relevant institutional, national, and international guidelines and legislation, with all necessary permissions and licenses obtained to ensure compliance with ethical and legal requirements.

### RNA extraction and reverse transcription polymerase chain reaction (RT-PCR)

We selected a total of 70 samples, including 10 asymptomatic and 60 symptomatic samples displaying symptoms such as necrotic ring blotch, brown rings, reddish-brown rings, leaf discoloration, albino, and chlorina. Approximately 100 mg of leaf tissue was ground in liquid nitrogen, and total RNA was extracted using the RiboEX Kit (GeneAll, South Korea), following the manufacturer’s instructions. The quality and quantity of total RNAs were assessed using NanoDrop (Thermo Scientific, USA). Complementary DNA (cDNA) was synthesized using the Easy cDNA Synthesis Kit (Parstous, Iran) with random hexamer primers, following the manufacturer’s protocol. PCR was performed using specific primers (TPNRBV1-F/TPNRBV1-R, TPNRBV2-F/TPNRBV2-R, TPNRBV3-F/TPNRBV3-R, and TPNRBV4-F/TPNRBV4-R) to amplify DNA fragments of 535 bp, 551 bp, 459 bp, and 407 bp, respectively, as described by Hao et al. (2018). Additionally, a pair of specific primers for the Movement Protein (MP) gene of TPNRBV was designed using Primer 3, based on RNA4 of TPNRBV (NC_040402.1) to amplify the complete MP segment (948 bp) for phylogenetic analyses and TPNRBV diagnosis. PCR conditions were optimized as follows: initial denaturation at 95 °C for 5 min, followed by 35 cycles of 95 °C for 30 s, annealing at the temperatures specified in Table 1 for 30 s, and extension at 72 °C for 40 s (for RNA segments) or 60 s (for MP), with a final extension at 72 °C for 7 min. PCR products were separated on 1% agarose gels stained with SYBR Safe (SinaClon, Iran) and visualized under UV light. Furthermore, the samples were screened for other known viruses infecting tea plants, including TPLPV, BNRBV, and APLPV.

**Table 1:** Characteristics of primer pairs used in this research.

Virus segment	Primer name	Sequence (5′ to 3′)	Ta (°C)	Size (bp)
TPNRBV RNA1	TPNRBV1-F	GCCCTGACAACGCAAAAGAACTGATG	50	535
	TPNRBV1-R	GTGACGGGATATTTTTGGACGACTGT		
TPNRBV RNA2	TPNRBV2-F	GGGCCGGGGTGTGGAAAAACTT	56	551
	TPNRBV2-R	TTCTTATCATCCCGGCAAAACACA		
TPNRBV RNA3	TPNRBV3-F	TTCGCCACTCACAAAGACAACAAACT	54	459
	TPNRBV3-R	GTAGCGGAGCGGAAAGAAAAGACT		
TPNRBV RNA4	TPNRBV4-F	TCAGTGGCGCGATTATCAGAAGGTA	58	407
	TPNRBV4-R	CGCGCAAGAAGTCGGTCAAAAC		
TPNRBV-MP	TPNRBV-MP-F	GCGGATCCATGTCTATAGC	48	948
	TPNRBV-MP-R	ACAAGCTTAAAAGTAATAGGTGGTACAGC		
BNRBV	BNRBV-F	GGTTTCGACACCTCGGCATG	56	560
	BNRBV-R	CCAGCTGCCCTTGAGACTTRTC		
TPLPV	TPLPV2-F	CCTATGGAGCTCTATGACGCAAATGAT	53	403
	TPLPV2-R	TCGACAGAGAAGTGATGGGGAAATAC		
APLPV	APLPV-F	GGTCGTCAAGGGAGAGGC	52	564
	APLPV-R	GGCCCCTAAGGGTCATTTC		

### Sequencing and phylogenetic analysis

We purified and directly sequenced 45 PCR products corresponding to the four RNA segments (RNA1-RNA4) of the TPNRBV genome for each of the nine isolates, as well as the MP gene of the same isolates. Sequencing was performed using the Sanger sequencing method (BGI Tech. Solutions, Shenzhen, China). The obtained sequences were edited and analyzed using BLASTn and were deposited in GenBank as novel TPNRBV sequences from Iran. Sequence alignment was carried out using ClustalW in MEGA 11 (Table 2)^20^. The best nucleotide substitution model for phylogenetic analysis was selected using MEGA 11, and phylogenetic trees were constructed using the Maximum Likelihood method with the GTR + G + I model and 1000 bootstrap replicates in MEGA 11 for RNA segments and MP sequences. Bootstrap values below 50% were omitted from the trees.

**Table 2:** List of isolates and their accession numbers of TPNRBV from this research and all other isolates.

Country	Isolate	Segment	GenBank accession no
Iran	A80, A20, A51, A1, B90, E50, P4, R10	RNA1	OK166790, OL739067, OL739068, OL739066, OK166793, OK166796, OK166802, OK166799, MW774643
		RNA2	OK166791, OL739070, OL739071, OL739069, OK166794, OK166797, OK166803, OK166800, MW774644
		RNA3	MZ476983, OL739072, OL739074, OL739076, MZ476984, MZ476985, MZ476986, MZ476987, MZ476981
		RNA4	OK166792, OL739073, OL739075, OL739077, OK166795, OK166798, OK166804, OK166801, MZ476982
	A80, A20, A1, E50, P4, R10, B49, Lahi, Otaghvar	MP	OM718722, ON380667, OM718719, OQ631080, OM718721, OM718720, OM718723, OQ589504, OQ589505
Japan	J	RNA 1	LC566237
		RNA2	LC566238
		RNA3	LC566239
		RNA4	LC566240
China	–	RNA 1	NC_040401, MG781152
		RNA2	NC_040404, MG781153
		RNA3	NC_040403, MG781154
		RNA4	NC_040402, MG781155

### Mechanical transmission of TPNRBV

To assess the mechanical transmission of TPNRBV, we selected two known isolates (R10 and A1) for assays. Approximately 100 mg of TPNRBV-infected leaf tissue was homogenized in a mortar with 1 mL of 0.1 M potassium phosphate buffer (pH 7.0). The sap was then directly applied to the leaves of *Nicotiana rustica* and *Chenopodium quinoa* seedlings at the three-four-leaf stage, previously treated with carborundum. Negative control seedlings were inoculated with buffer alone. Inoculated plants were kept under controlled conditions in a greenhouse (temperature 22 °C with a 14:10 h light/dark photoperiod) and monitored daily for symptom development. This experiment was repeated twice, with ten replicates of each plant species per experiment. At 30–35 days post-inoculation (dpi), total RNA was extracted from inoculated plants using the cetyltrimethylammonium bromide (CTAB) method^21^, and the presence of TPNRBV was confirmed by RT-PCR using specific primers, as described above, followed by sequencing.

### Seed transmission of TPNRBV

We conducted experiments to investigate whether TPNRBV could infect tea seeds. Seeds were collected from virus-infected plants displaying symptoms in late autumn 2022. The possibility of TPNRBV seed transmission was examined by growing the collected seeds in a virus-free greenhouse. Forty seeds obtained from infected plants were sown in pots containing suitable soil and covered with plastic sheets to maintain warmth and moisture. These pots were placed in an insect-proof greenhouse under optimal conditions for tea plant growth. Seedlings were transferred to new pots (five seedlings per pot) when buds emerged in April. Weekly inspections were conducted to record any symptoms. Finally, in late September 2022, leaves were collected from the pots to detect TPNRBV using RT-PCR, and the amplified fragments from seedlings were sequenced.

Additionally, to detect TPNRBV in different parts of the seeds (embryo, cotyledon, and seed coat), we collected 100 seeds from virus-infected plants. The seeds were randomly divided into ten subgroups, each containing ten seeds, and total RNA was extracted. Subsequently, seeds were dissected, and fine-point forceps soaked in 70% ethanol was used to separate embryos from cotyledons. Embryos, coats, and cotyledons from each subgroup were mixed, and total RNA was extracted. The extracted total RNA from each subgroup was independently subjected to cDNA synthesis, followed by PCR using a specific primer pair (TPNRBV3-F/TPNRBV3-R) to determine the presence of TPNRBV in different parts of the seed.

Moreover, we collected several symptomatic pods from plants confirmed to be infected with TPNRBV. The pods were washed, and disinfected with sterile water and 70% ethanol, and total nucleic acids were extracted from two sample groups, each consisting of five symptomatic pods. Subsequently, RT-PCR was optimized to detect TPNRBV using specific primers.

### Ethical approval

This article does not contain any studies with human or animal subjects performed by any of the authors.

### Plant identification

The plant species used in this study were identified by Dr. Bahrami, a plant taxonomist at the University of Maragheh. The taxonomic identification followed established botanical literature and expert knowledge in the field.

### Supplementary Information

Supplementary Information 1.

Supplementary Information 2.

Supplementary Information 3.

## Data Availability

All data generated and analyzed during this study are included in this published article and sequences were deposited in NCBI-GenBank with accession numbers, OK166790, OK166791, MZ476983, OK166792, OL739067, OL739070, OL739072, OL739073, OL739068, OL739071, OL739074, OL739075, OL739066, OL739069, OL739076, OL739077, OK166793, OK166794, MZ476984, OK166795, OK166796, OK166797, MZ476985, OK166798, OK166802, OK166803, MZ476986, OK166804, OK166799, OK166800, MZ476987, OK166801, MW774643, MW774644, MZ476981, MZ476982, OM718722, ON380667, OM718719, OQ631080, OM718721, OM718720, OM718723, OQ589504, OQ589505.
