# Supplementary material for: Molecular and biological investigating of tea plant necrotic ring blotch virus as a worldwide threat
Source: Sci Rep. 2023 Nov 4;13:19113. doi: 10.1038/s41598-023-46654-3 (PMC10625587; doi:10.1038/s41598-023-46654-3)
Supplement: Supplementary file 1 — Supplementary Information 1. [file 41598_2023_46654_MOESM1_ESM.pptx]

## Slide 1
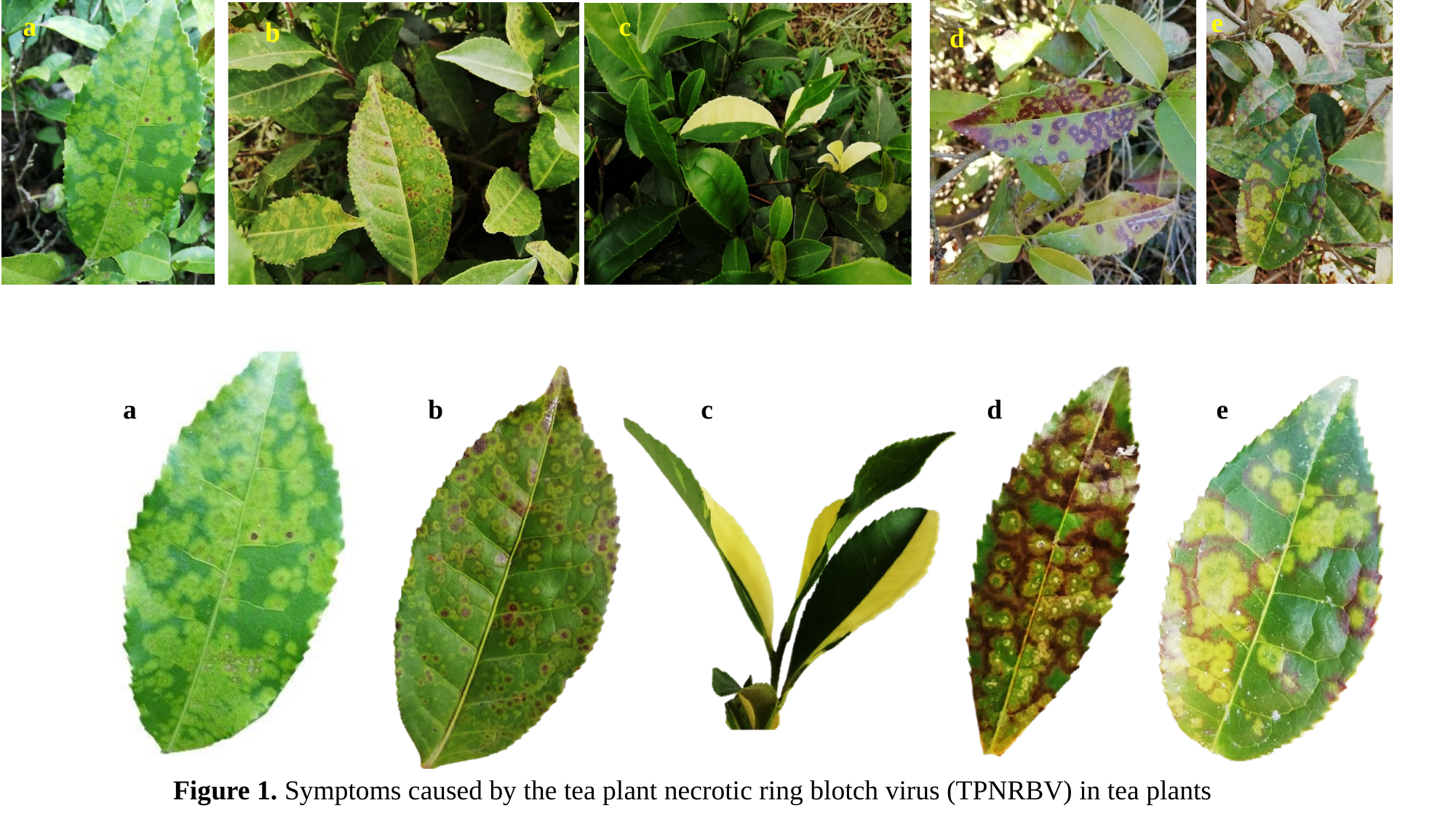

e
a
c
b
d
a
b
c
d
e
Figure 1. Symptoms caused by the tea plant necrotic ring blotch virus (TPNRBV) in tea plants

## Slide 2
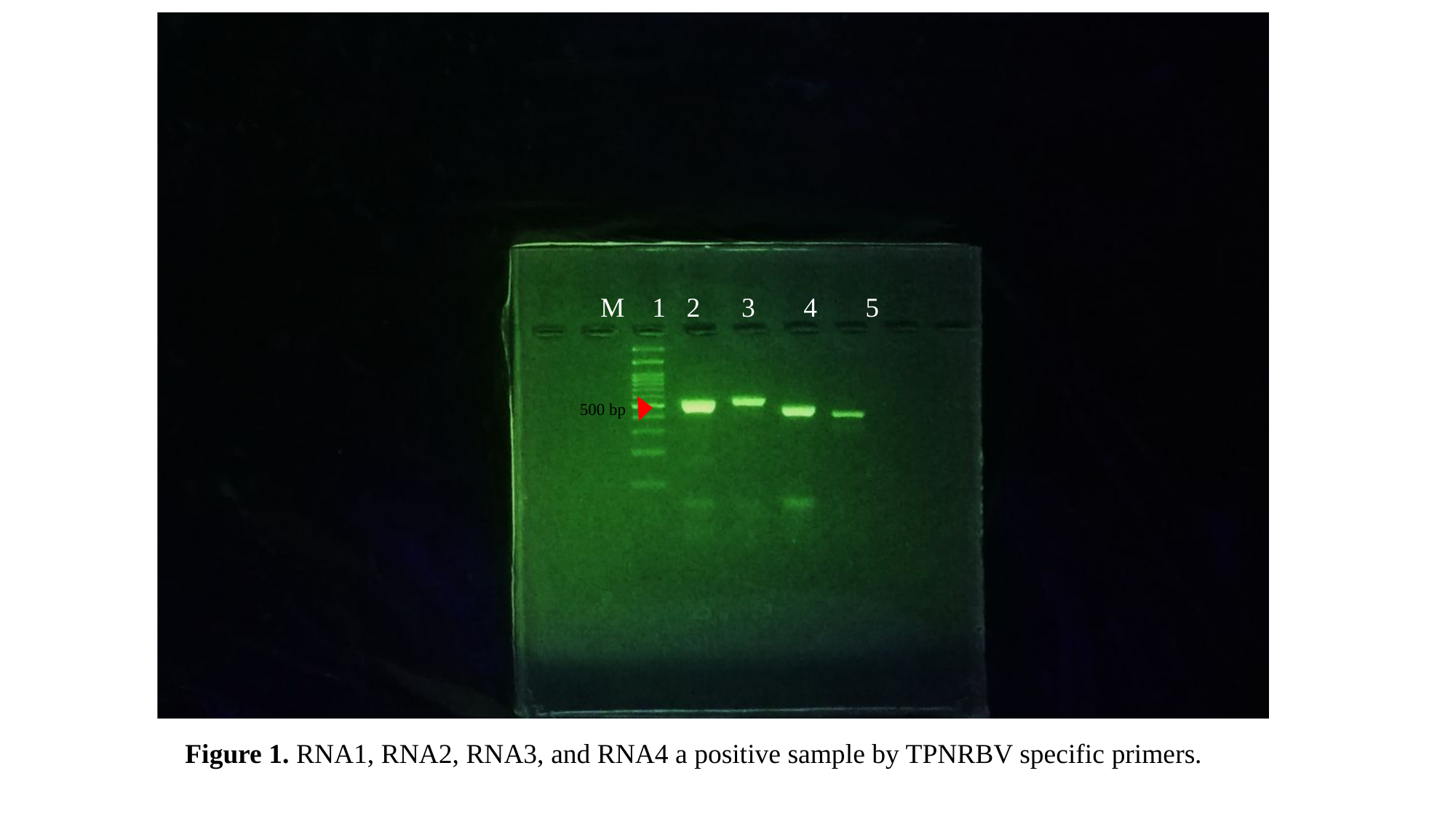

M 1 2 3 4 5
500 bp
Figure 1. RNA1, RNA2, RNA3, and RNA4 a positive sample by TPNRBV specific primers.

## Slide 3
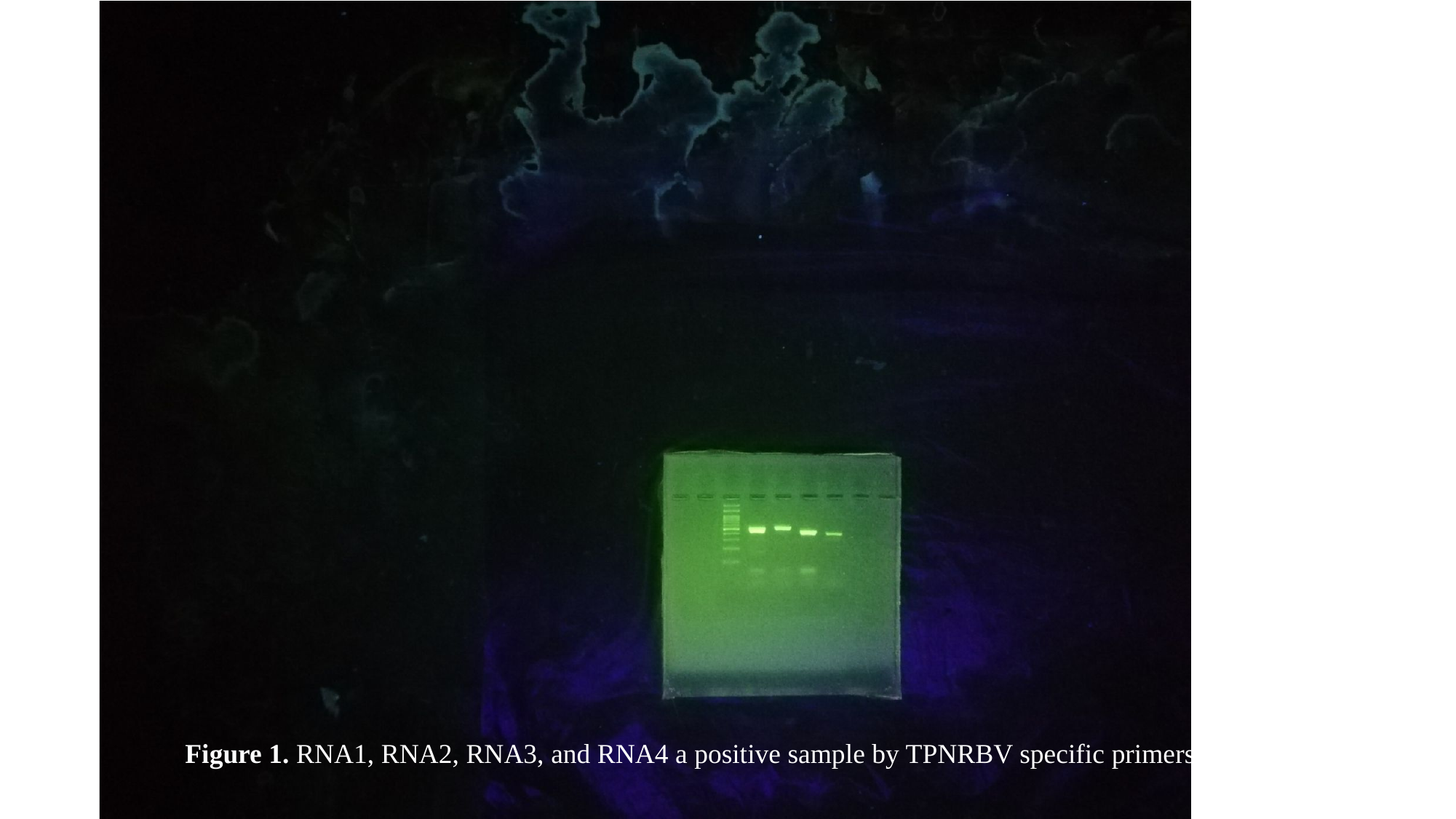

Figure 1. RNA1, RNA2, RNA3, and RNA4 a positive sample by TPNRBV specific primers.

## Slide 4
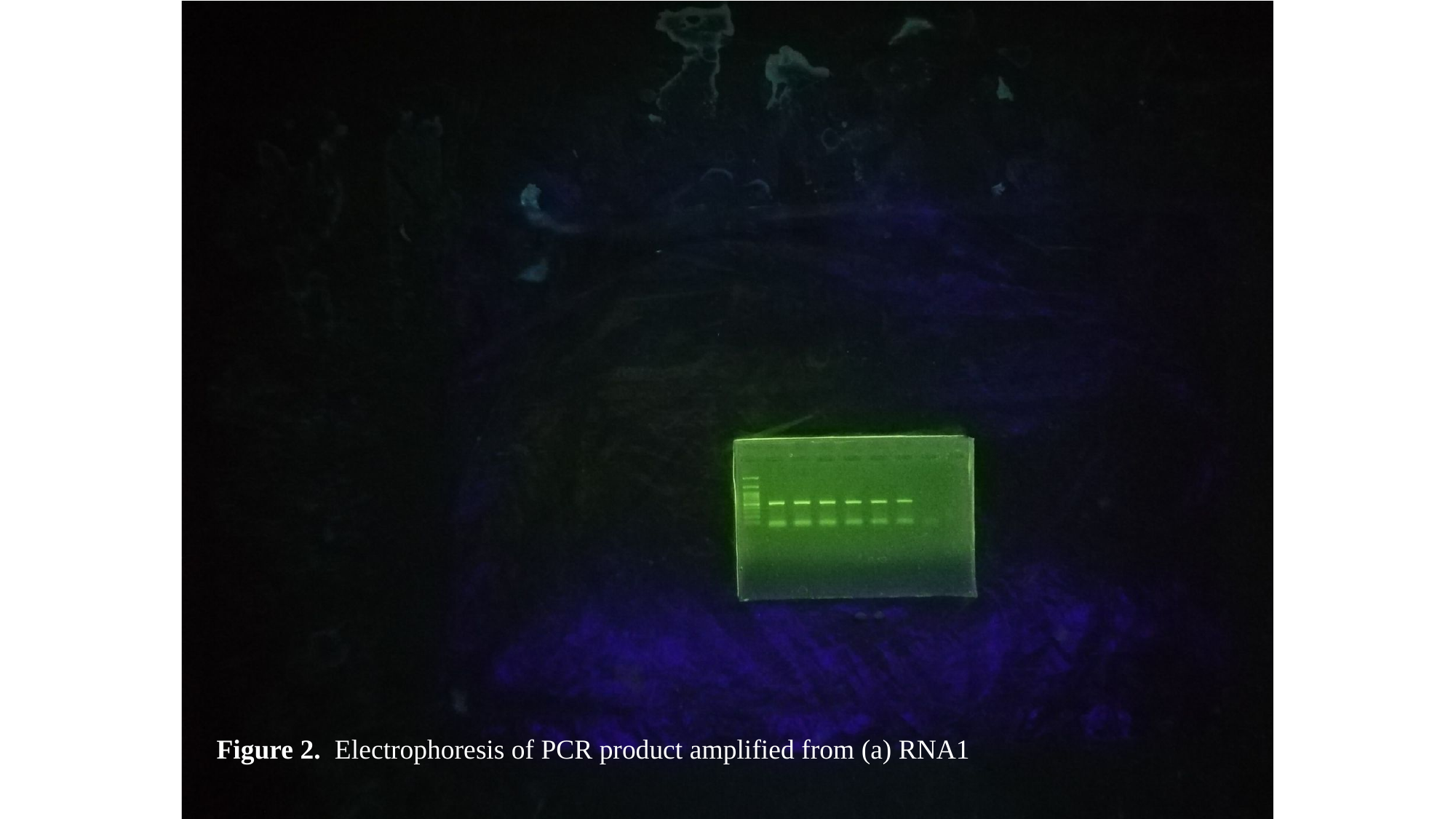

Figure 2. Electrophoresis of PCR product amplified from (a) RNA1

## Slide 5
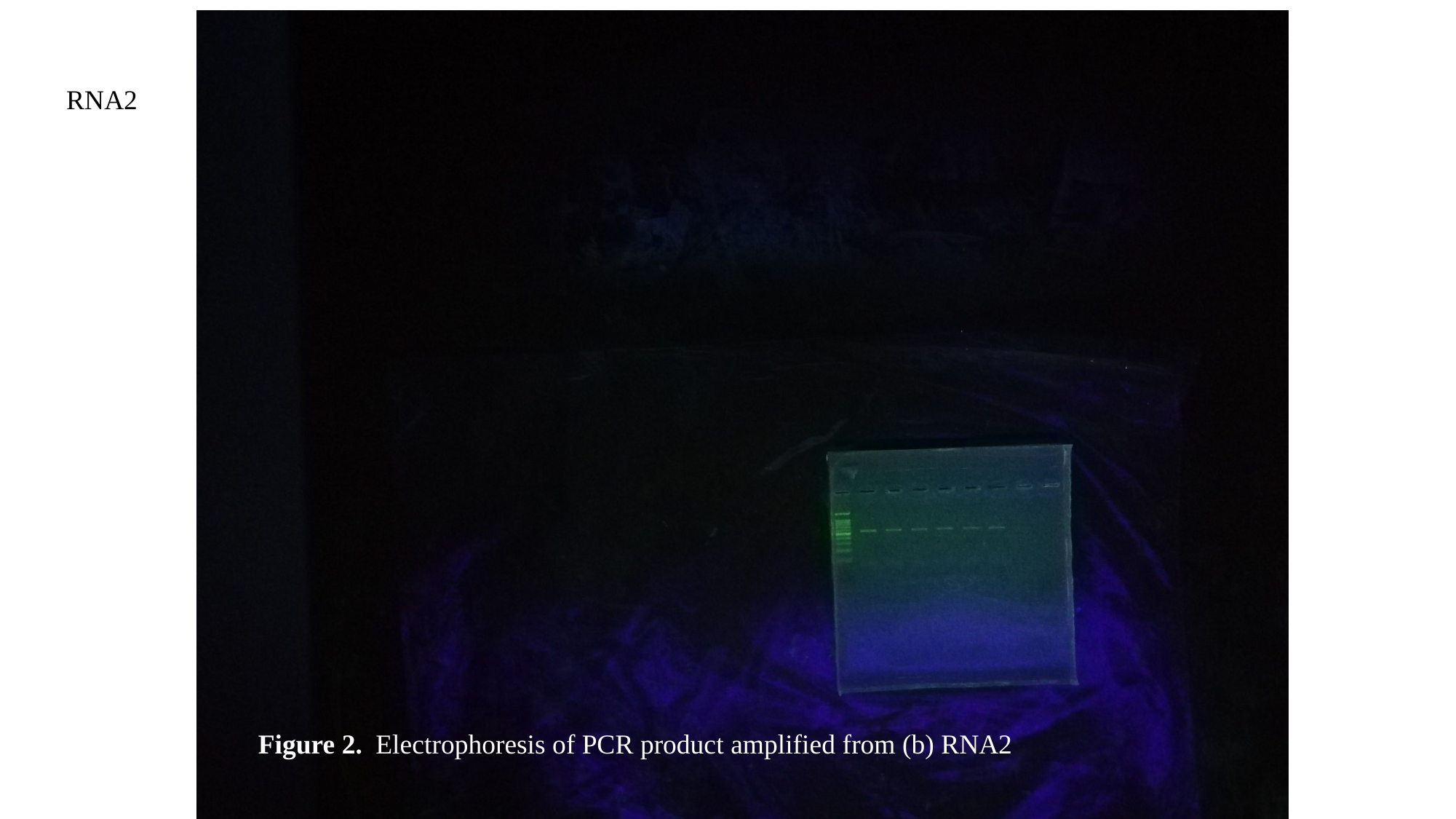

RNA2
Figure 2. Electrophoresis of PCR product amplified from (b) RNA2

## Slide 6
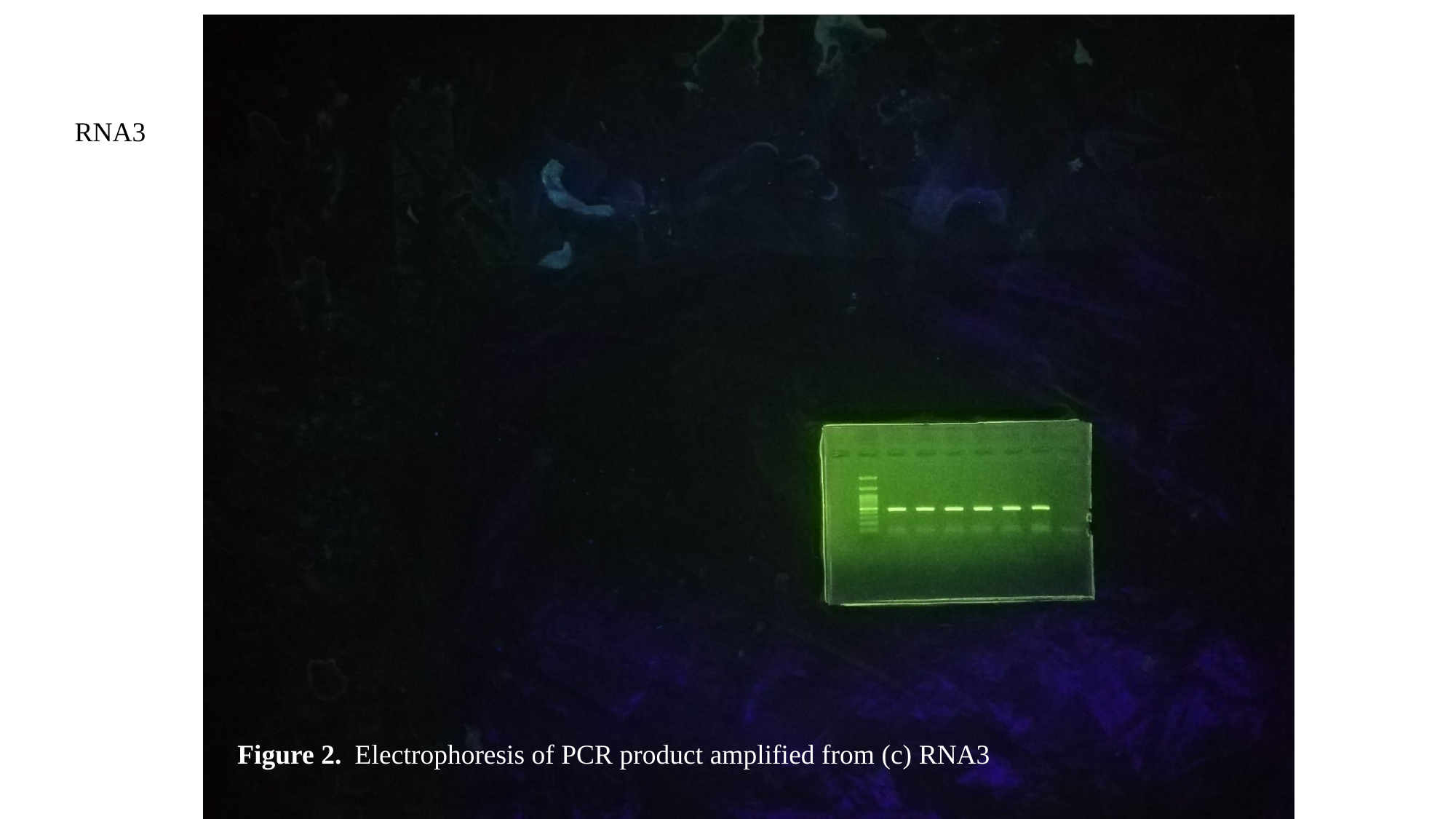

RNA3
Figure 2. Electrophoresis of PCR product amplified from (c) RNA3

## Slide 7
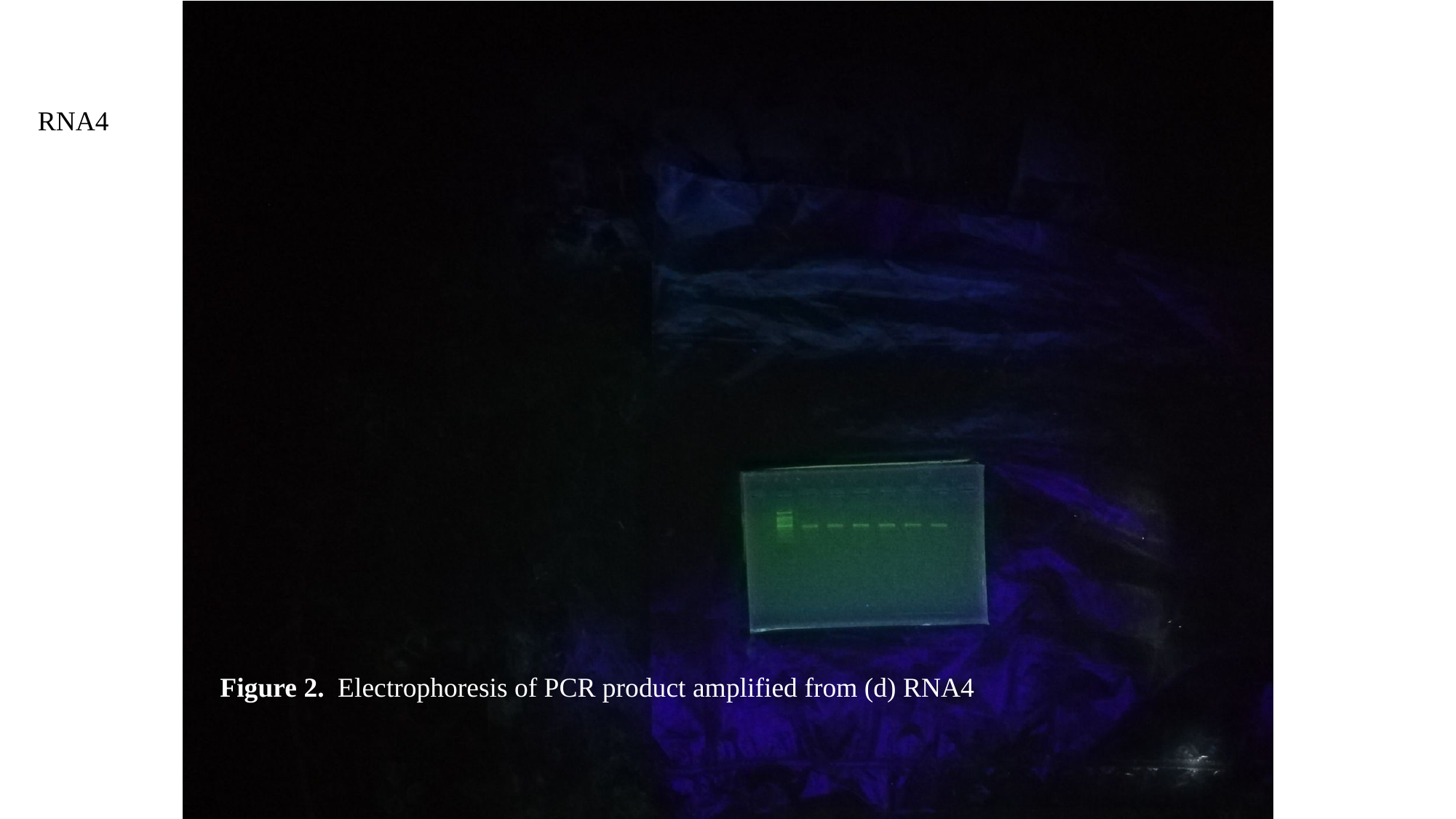

RNA4
Figure 2. Electrophoresis of PCR product amplified from (d) RNA4

## Slide 8
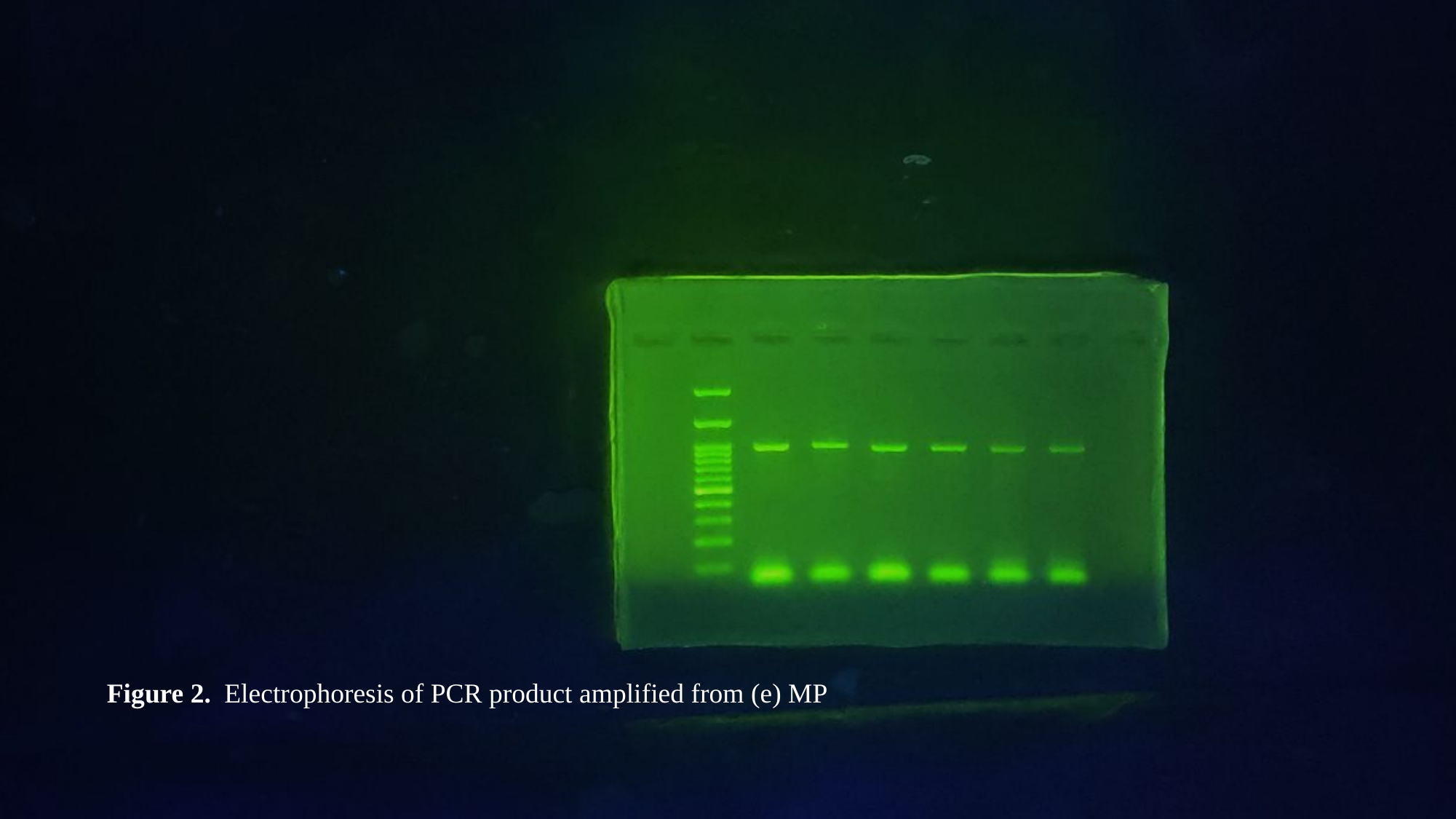

Figure 2. Electrophoresis of PCR product amplified from (e) MP

## Slide 9
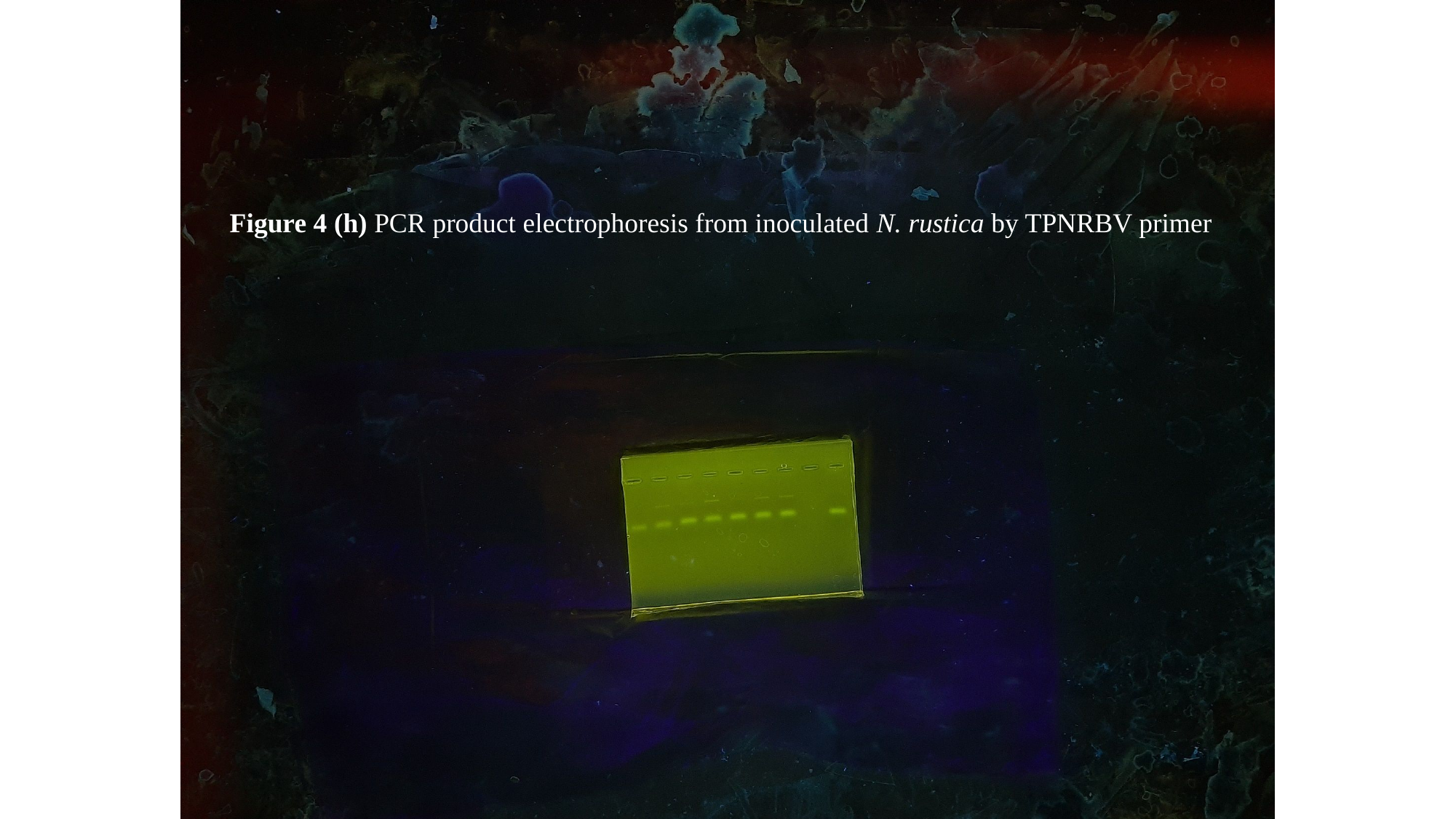

Figure 4 (h) PCR product electrophoresis from inoculated N. rustica by TPNRBV primer

## Slide 10
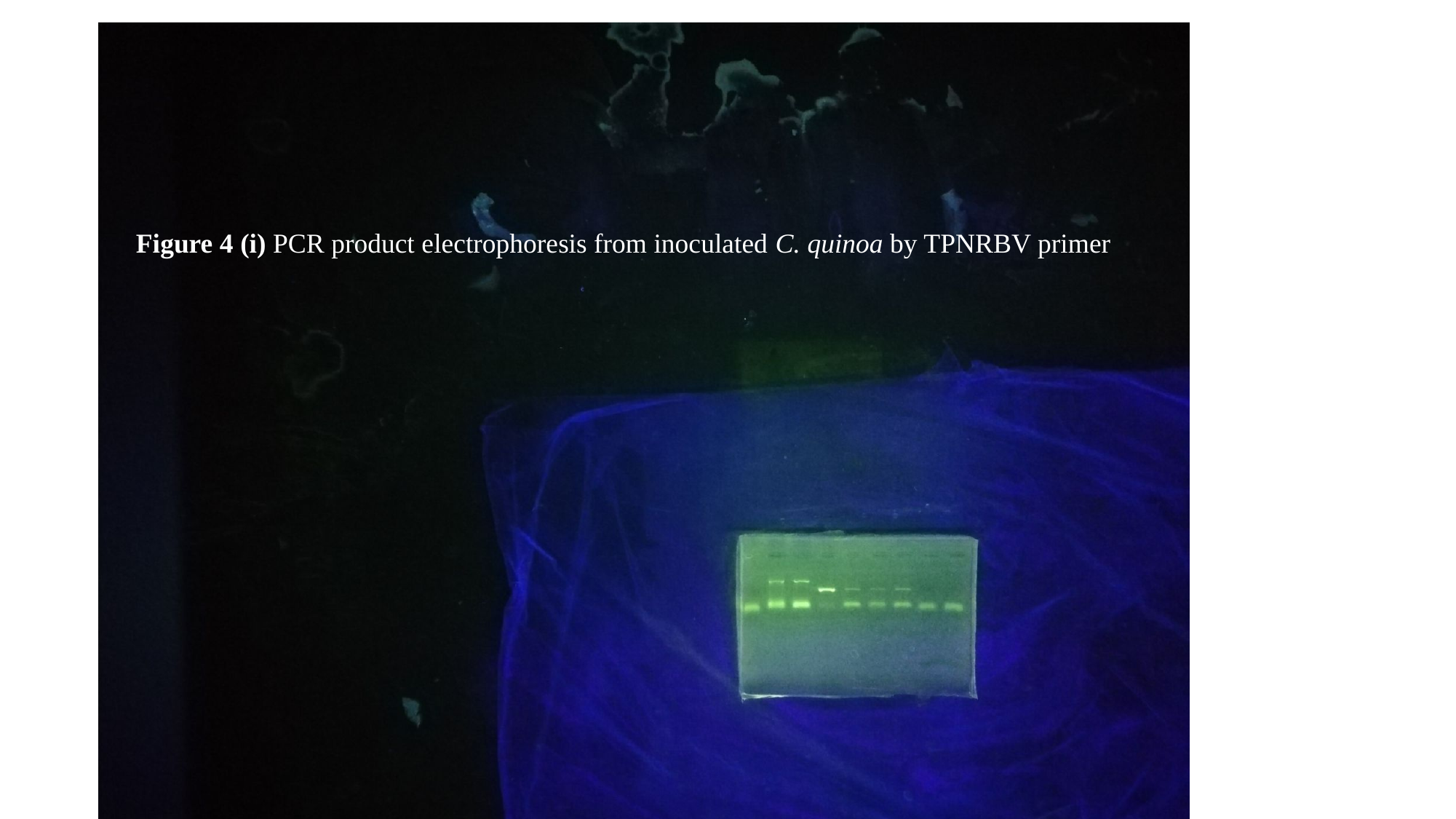

Figure 4 (i) PCR product electrophoresis from inoculated C. quinoa by TPNRBV primer

## Slide 11
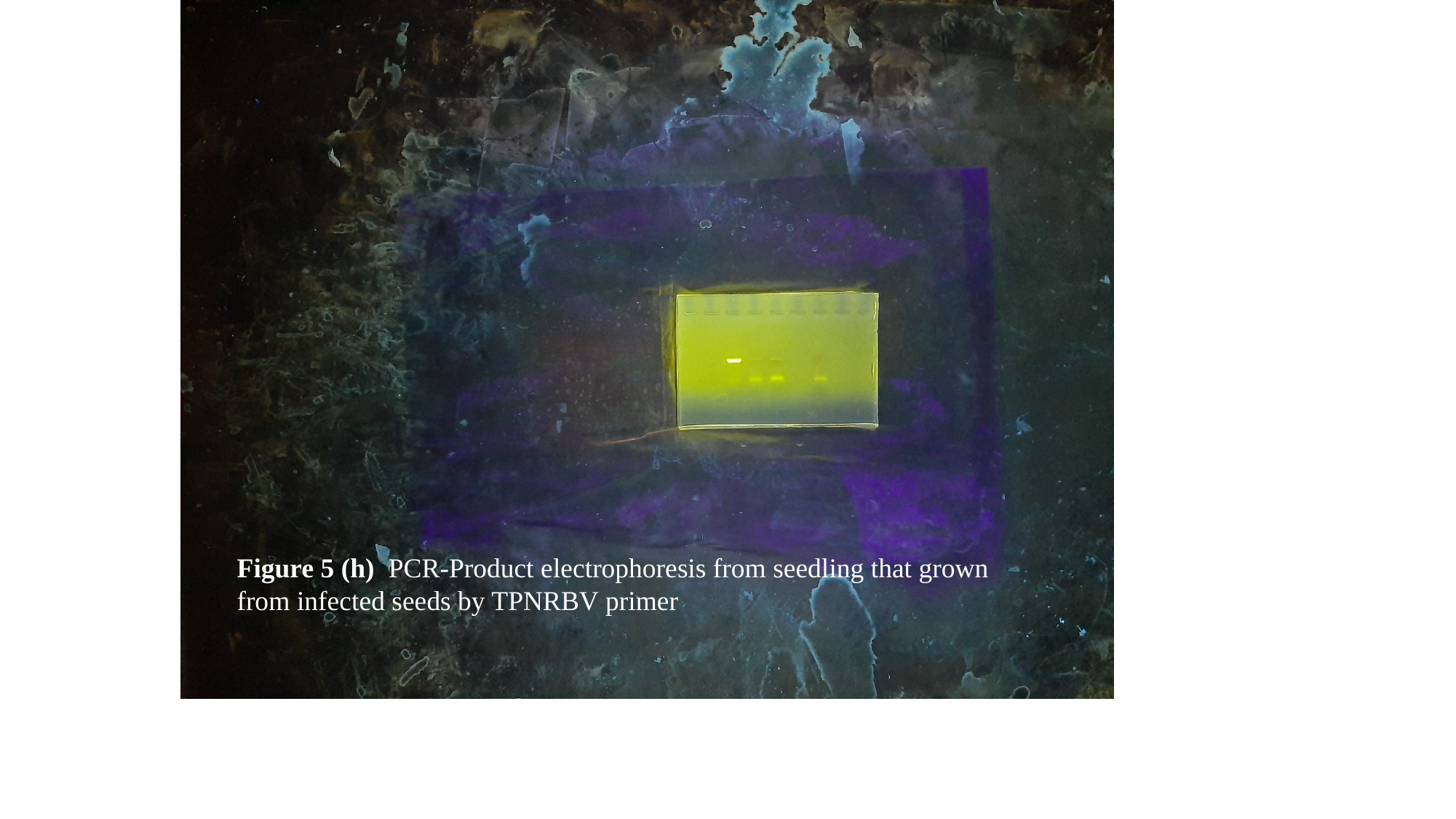

Figure 5 (h) PCR-Product electrophoresis from seedling that grown from infected seeds by TPNRBV primer

## Slide 12
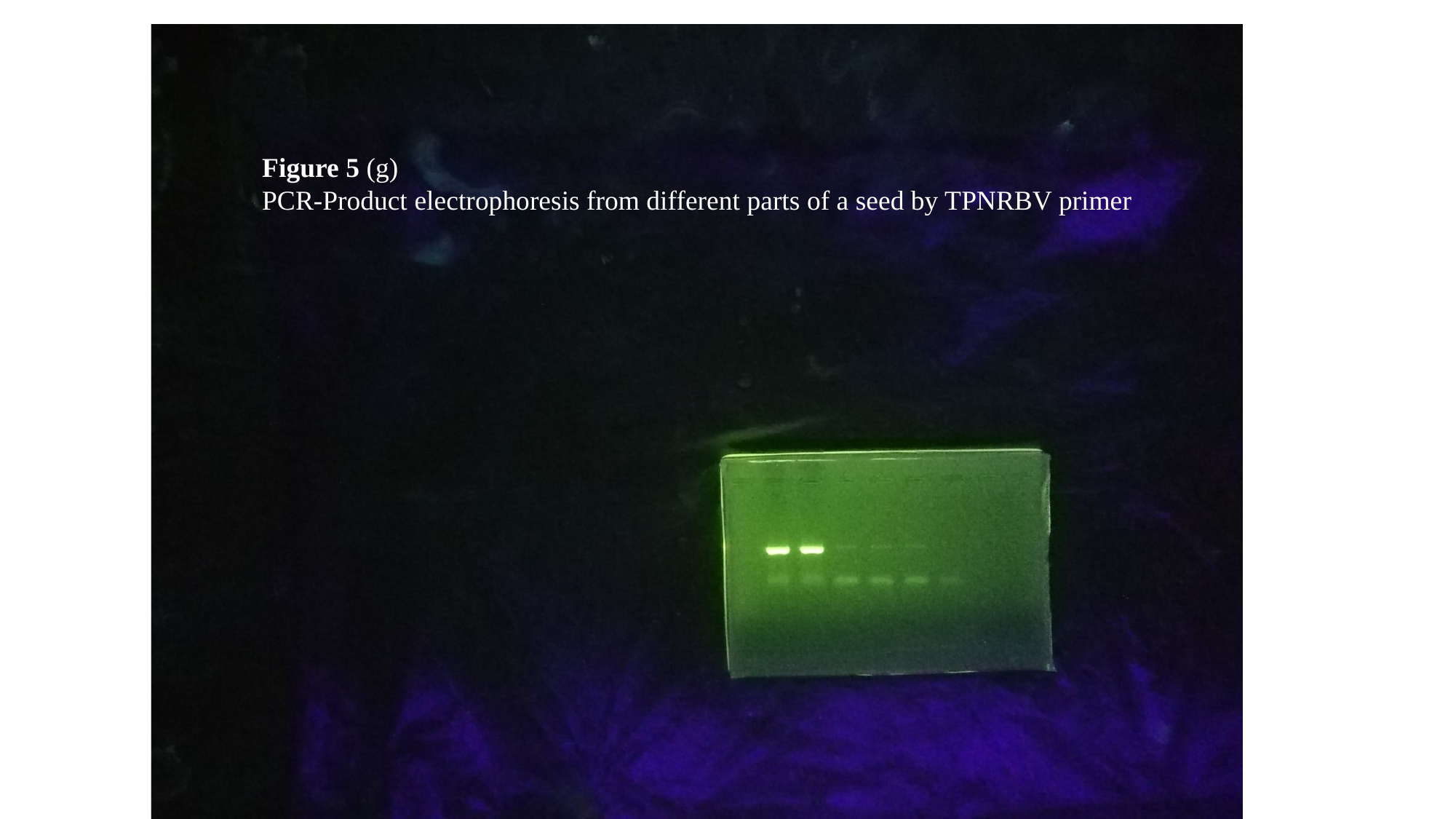

Figure 5 (g)
PCR-Product electrophoresis from different parts of a seed by TPNRBV primer
