# Supplementary material for: Molecular and biological investigating of tea plant necrotic ring blotch virus as a worldwide threat
Source: Sci Rep. 2023 Nov 4;13:19113. doi: 10.1038/s41598-023-46654-3 (PMC10625587; doi:10.1038/s41598-023-46654-3)

## Slide 1
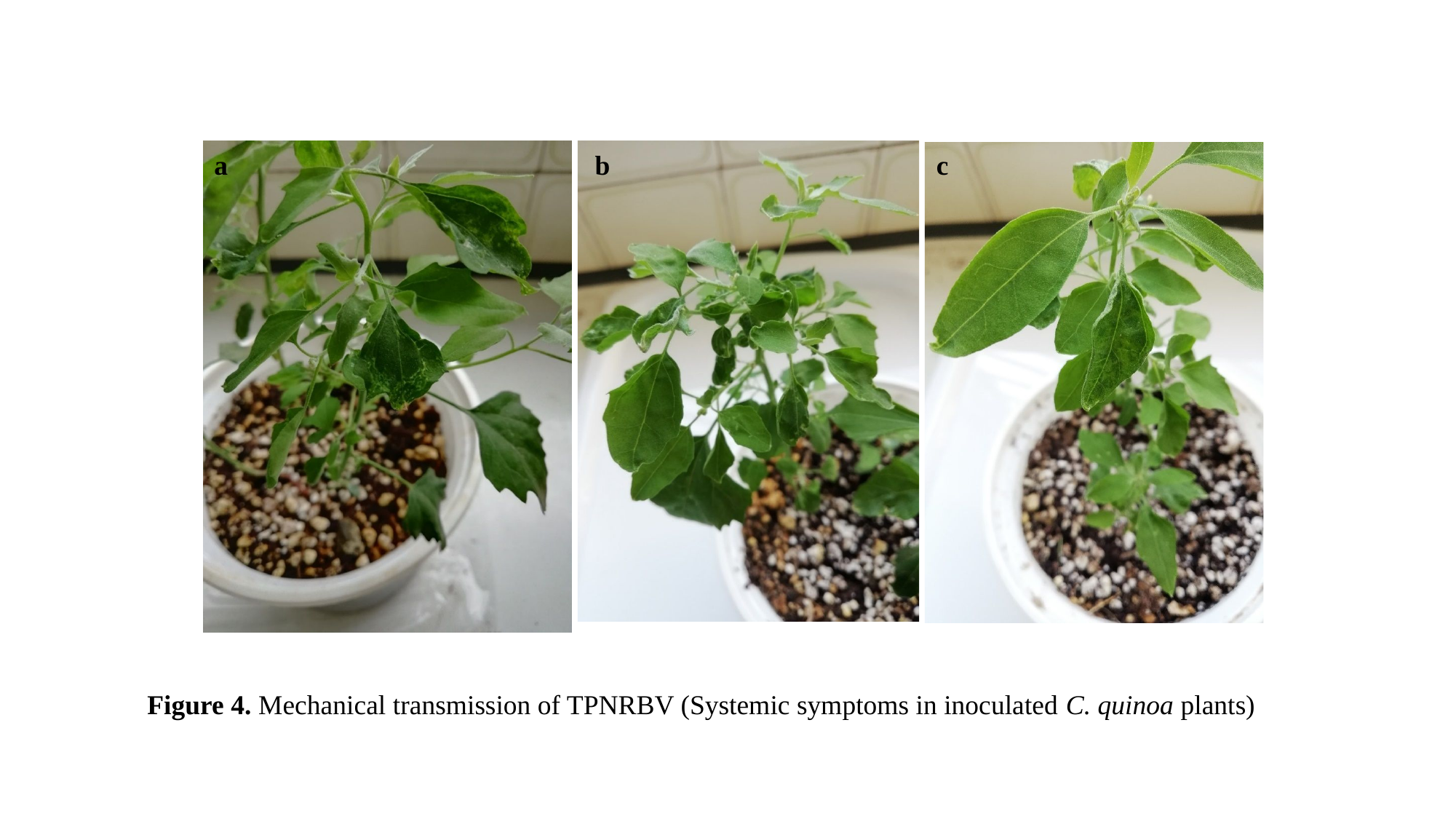

a
c
b
Figure 4. Mechanical transmission of TPNRBV (Systemic symptoms in inoculated C. quinoa plants)

## Slide 2
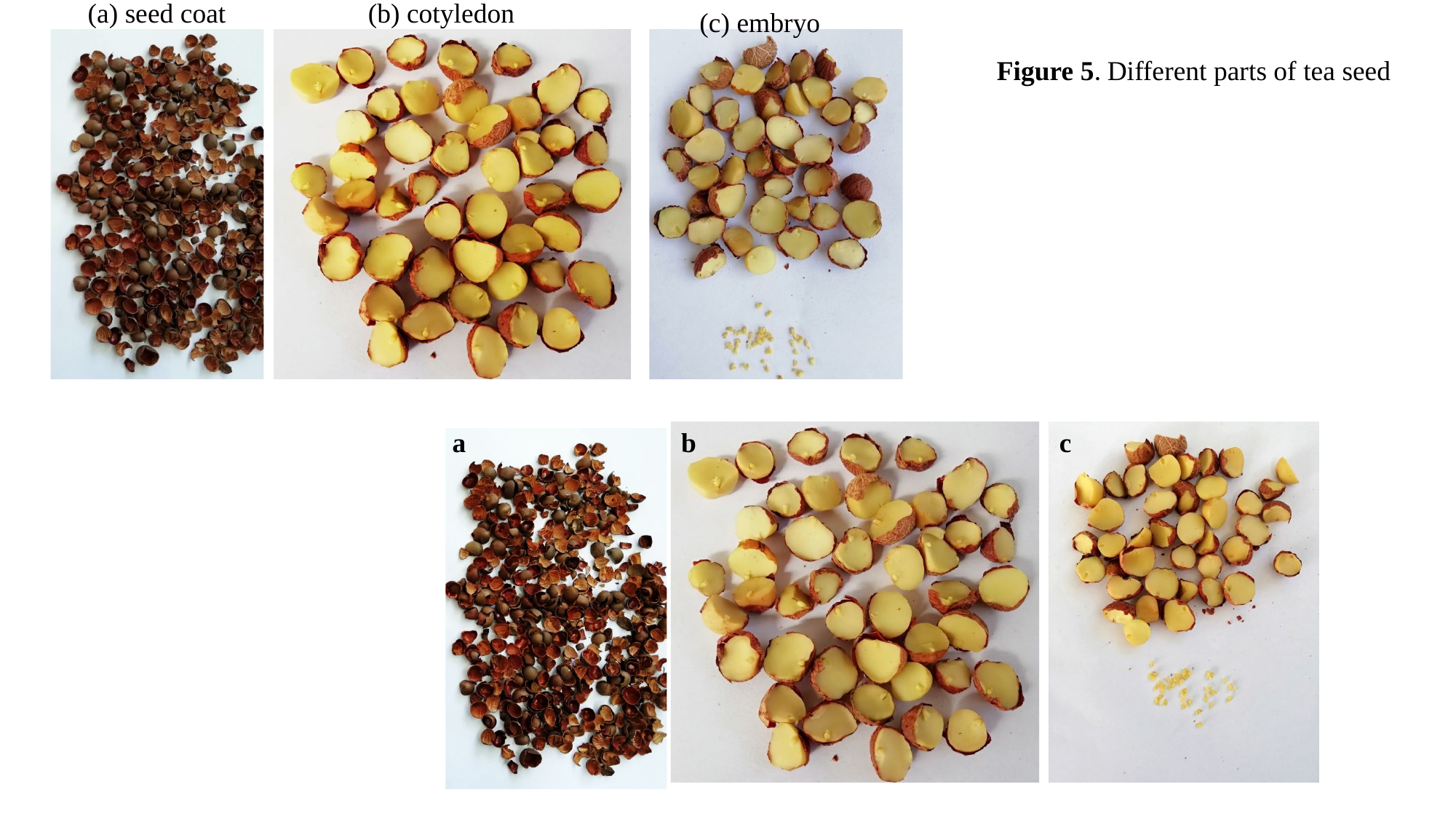

(c) embryo
(a) seed coat
(b) cotyledon
Figure 5. Different parts of tea seed
a
b
c

## Slide 3
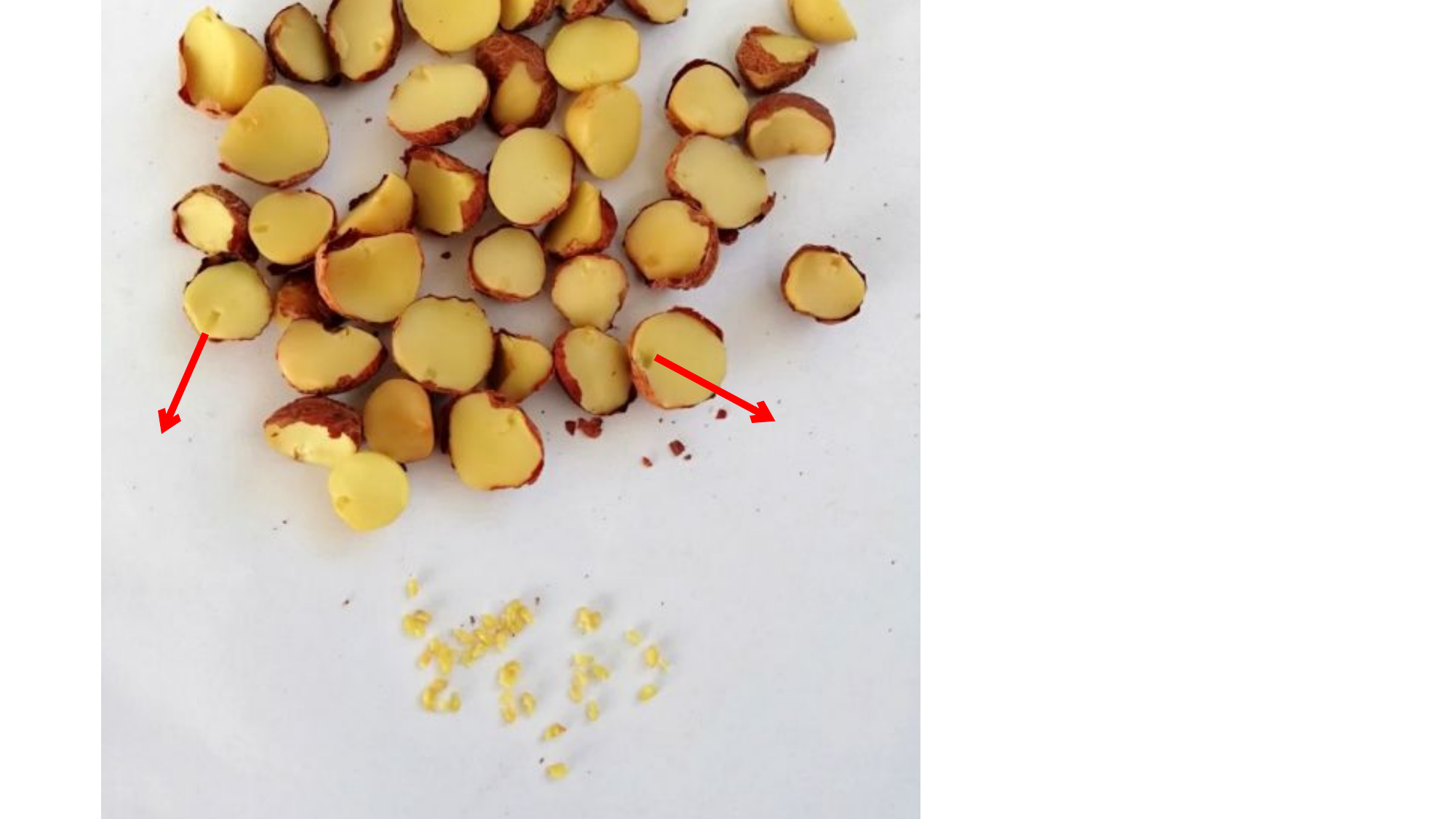

جنین جدا شده

## Slide 4
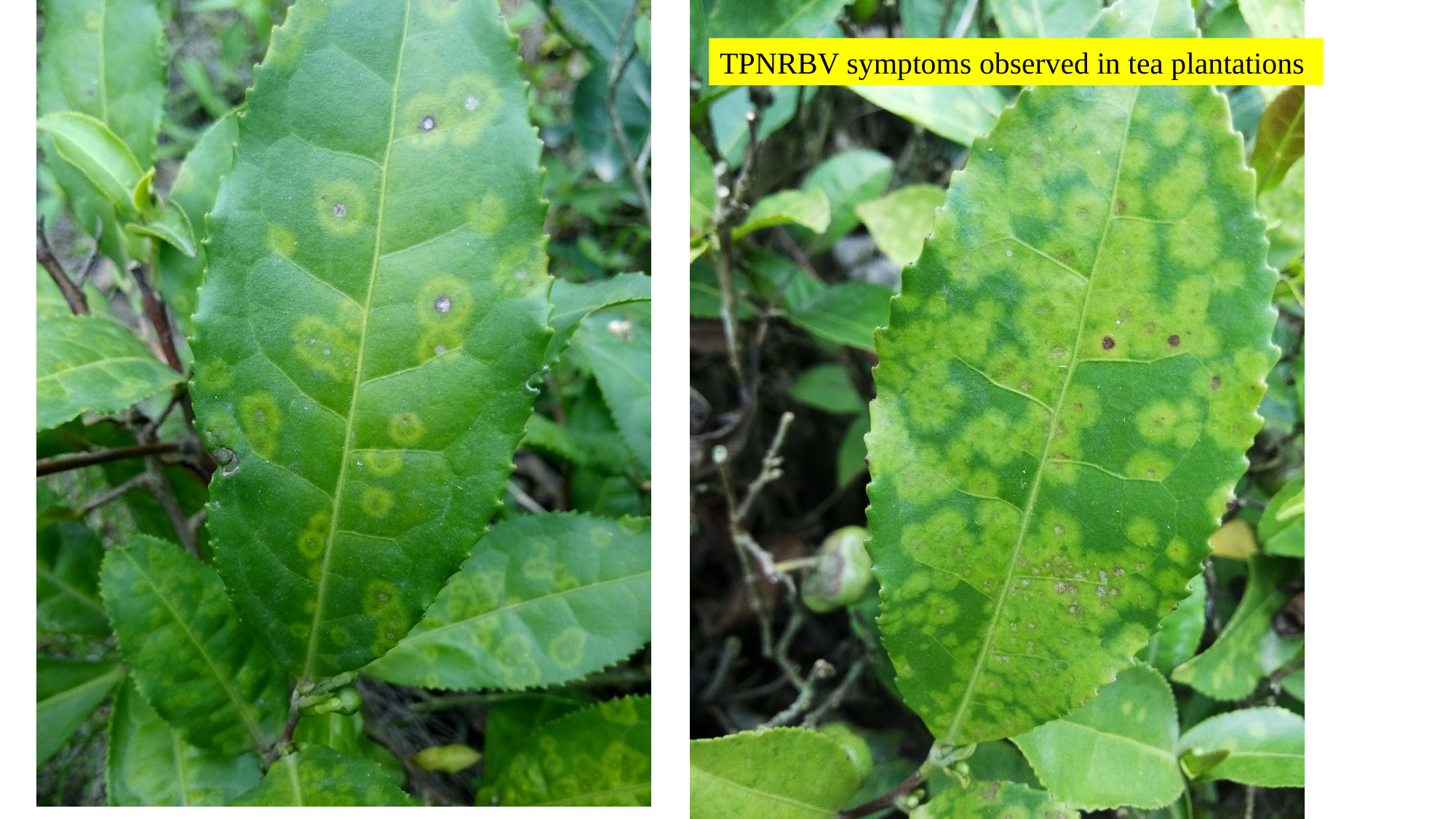

TPNRBV symptoms observed in tea plantations

## Slide 5
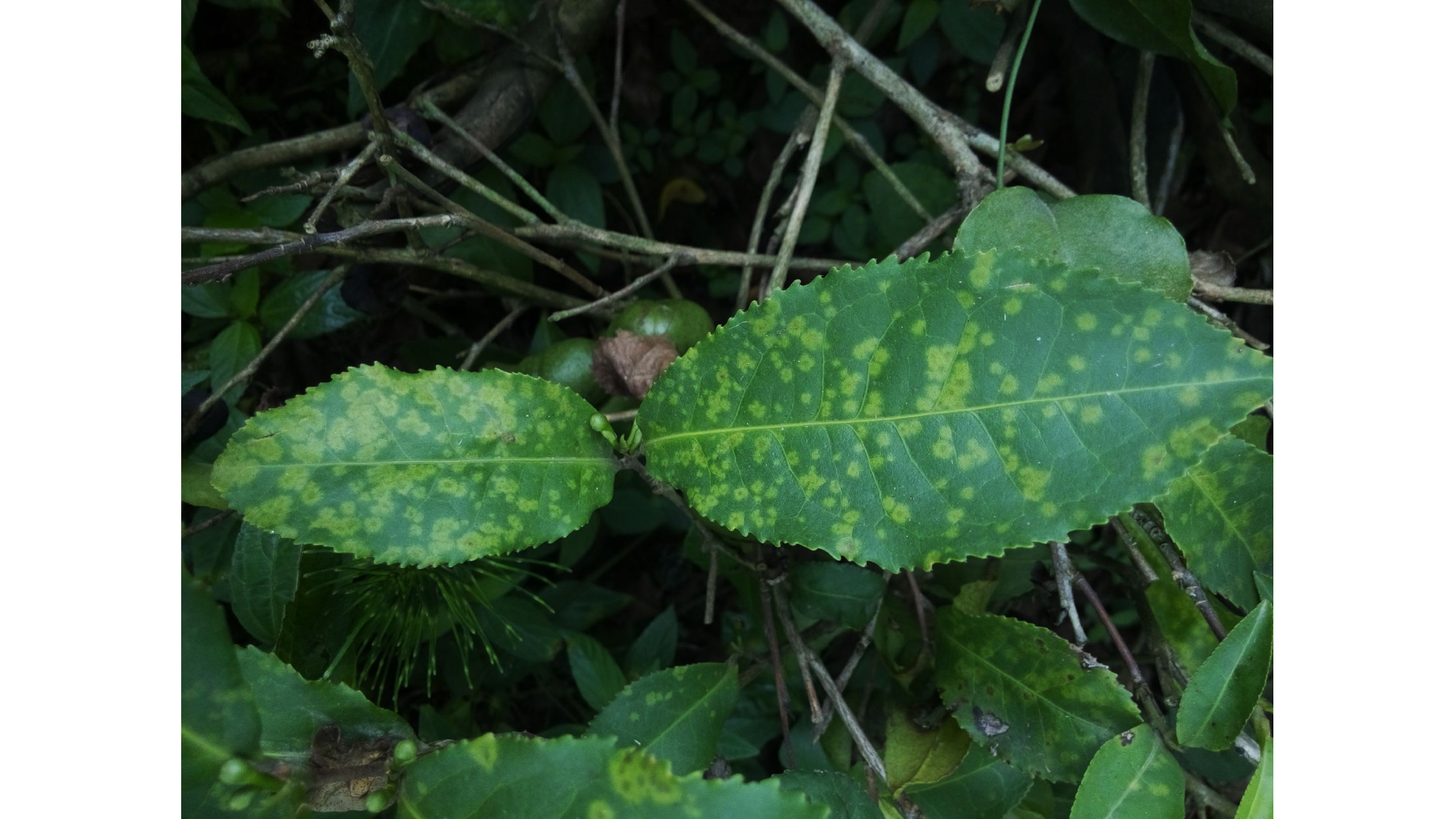

## Slide 6
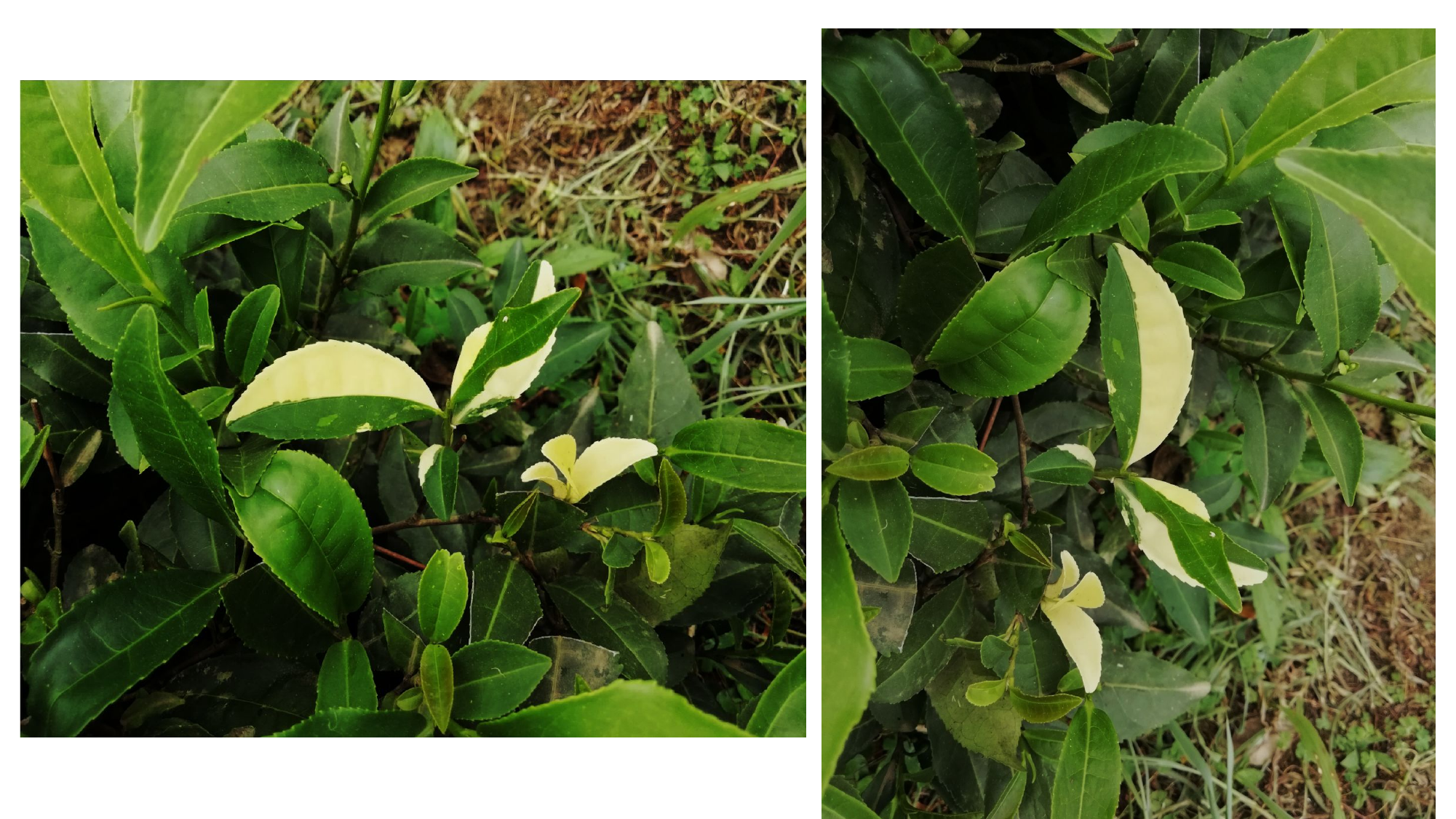

## Slide 7
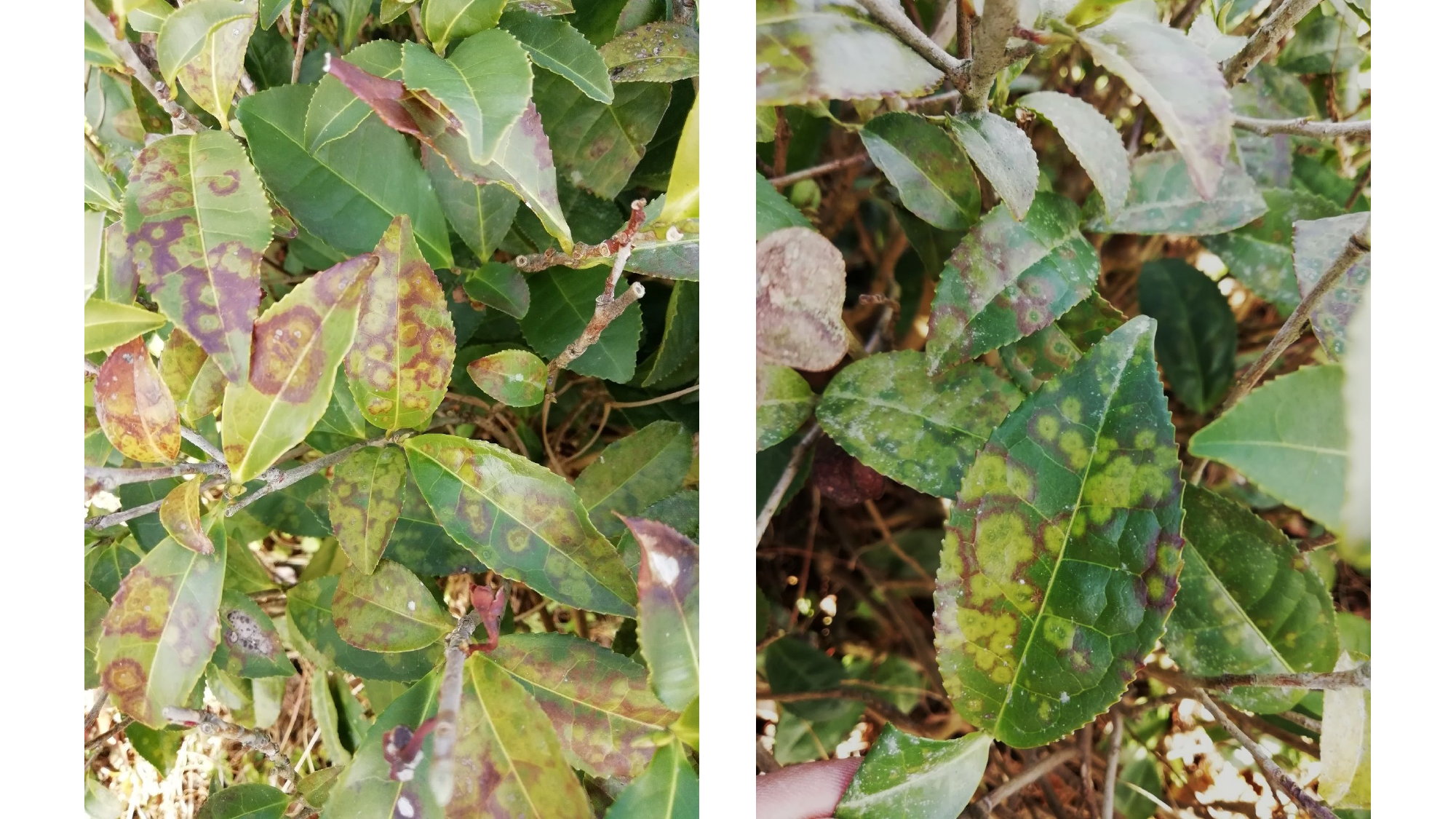

## Slide 8
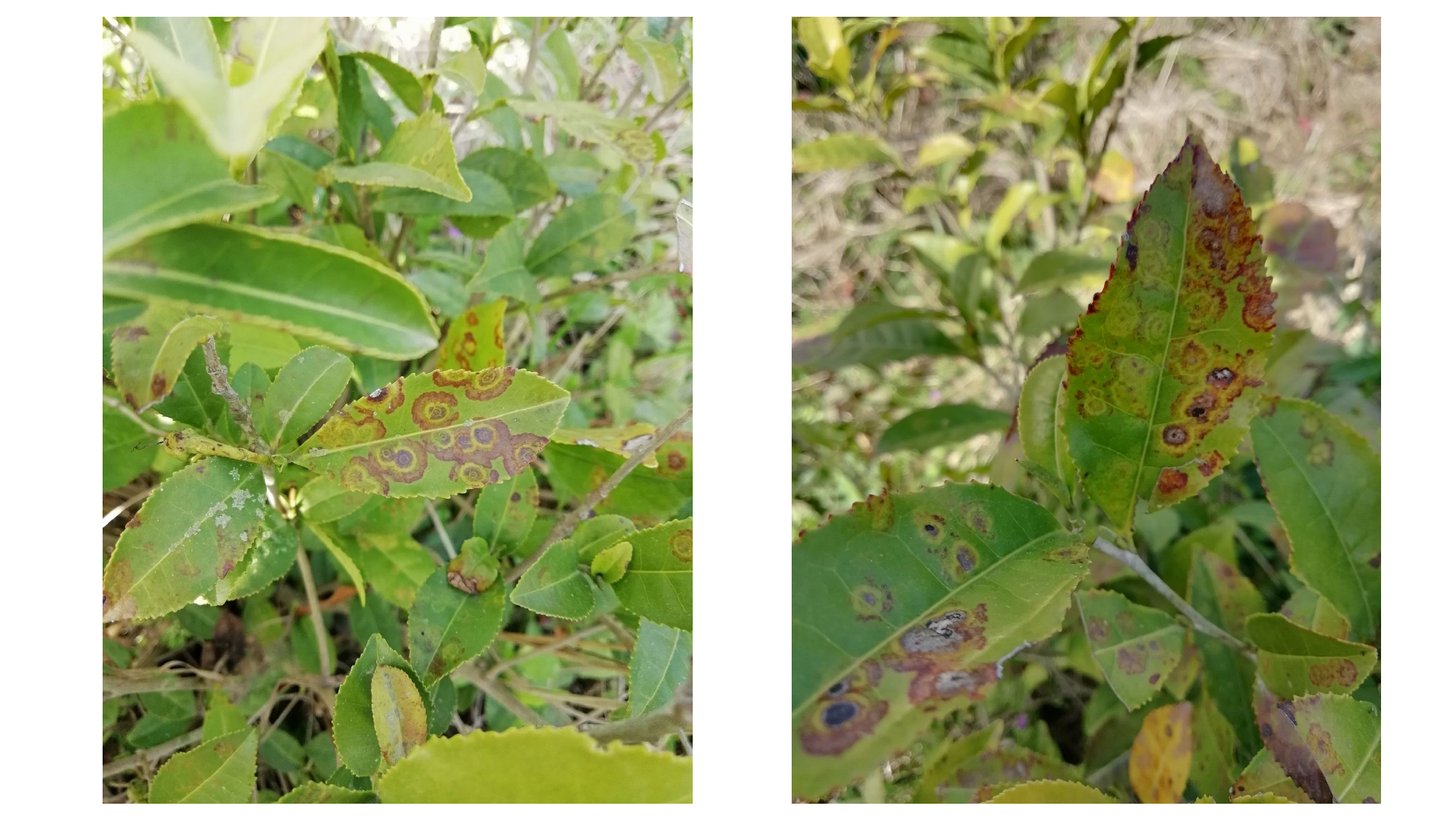

## Slide 9
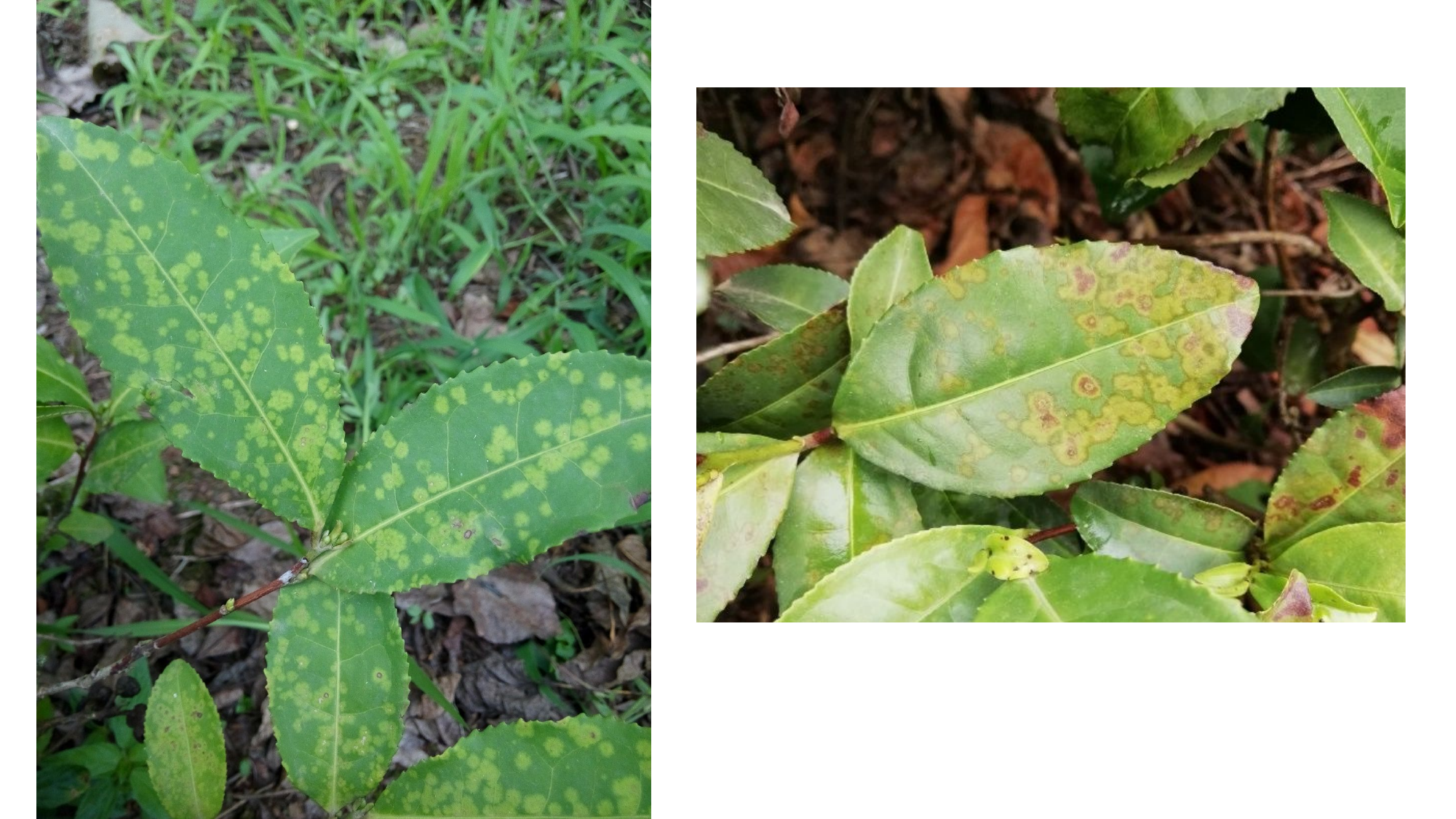

## Slide 10
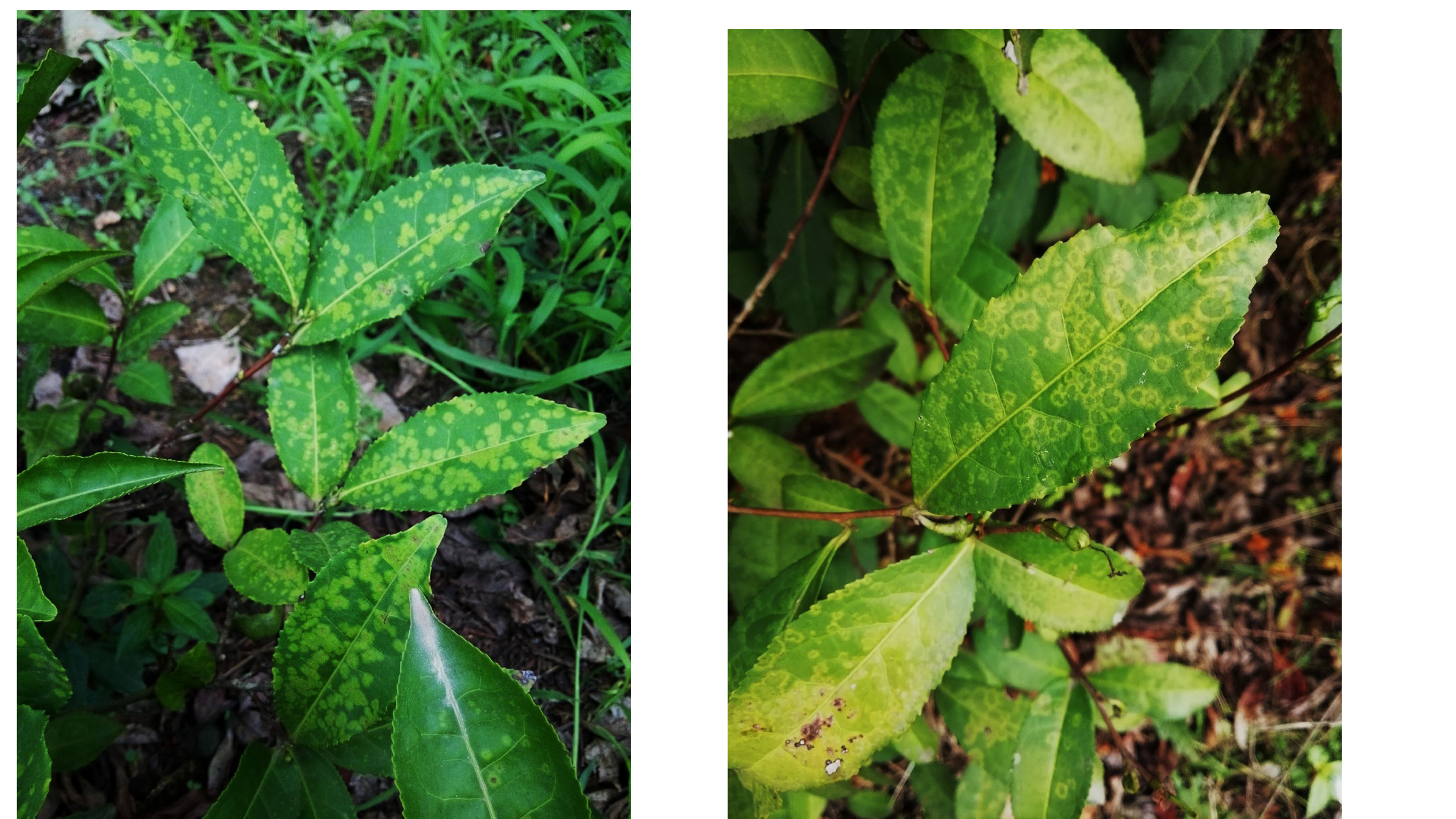

Supplement: Supplementary file 2 — Supplementary Information 2. [file 41598_2023_46654_MOESM2_ESM.pptx]
