# Supplementary figures and images for: Molecular and biological investigating of tea plant necrotic ring blotch virus as a worldwide threat
Source: Sci Rep. 2023 Nov 4;13:19113. doi: 10.1038/s41598-023-46654-3 (PMC10625587; doi:10.1038/s41598-023-46654-3)

## Slide 1
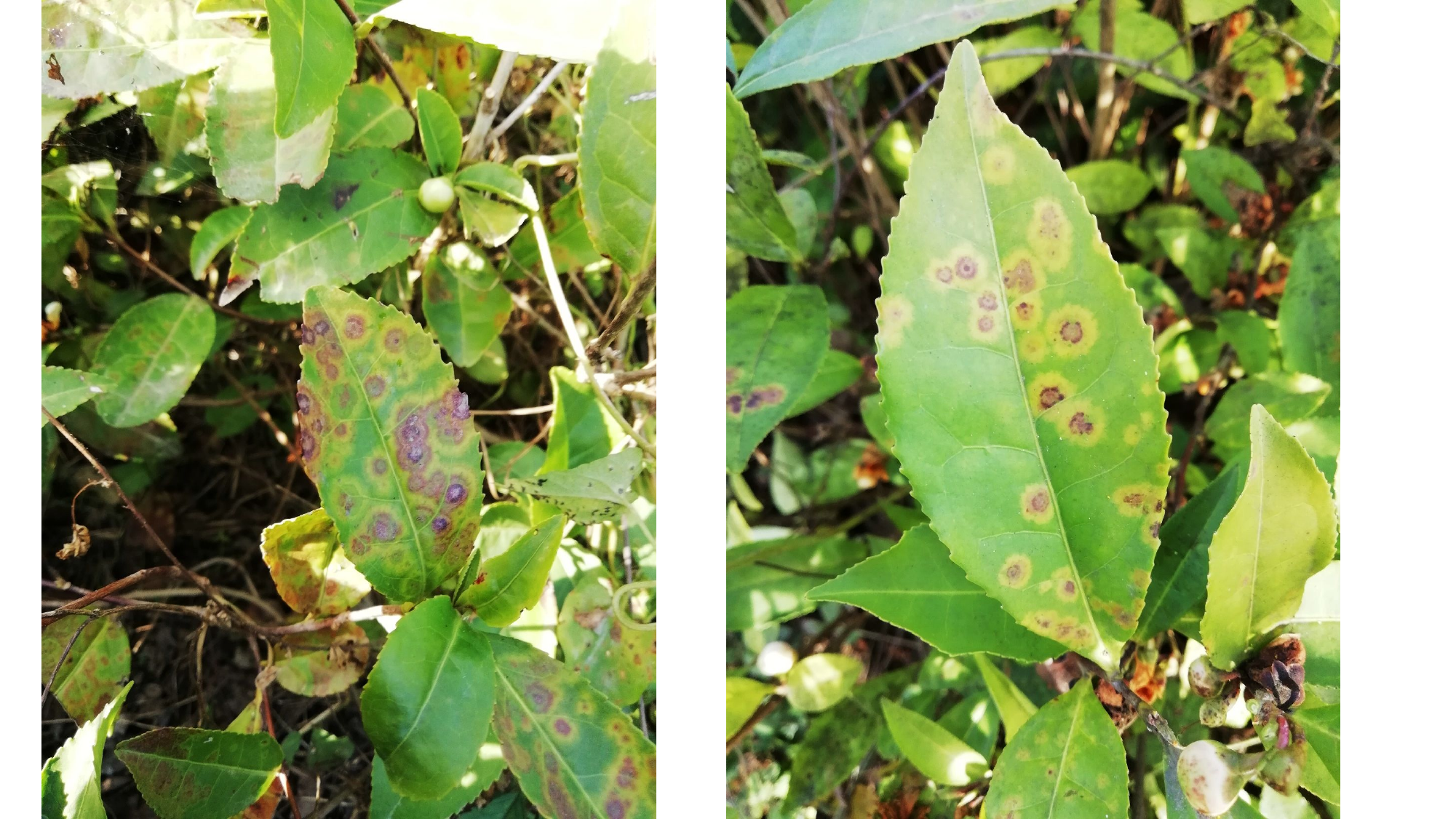

## Slide 2
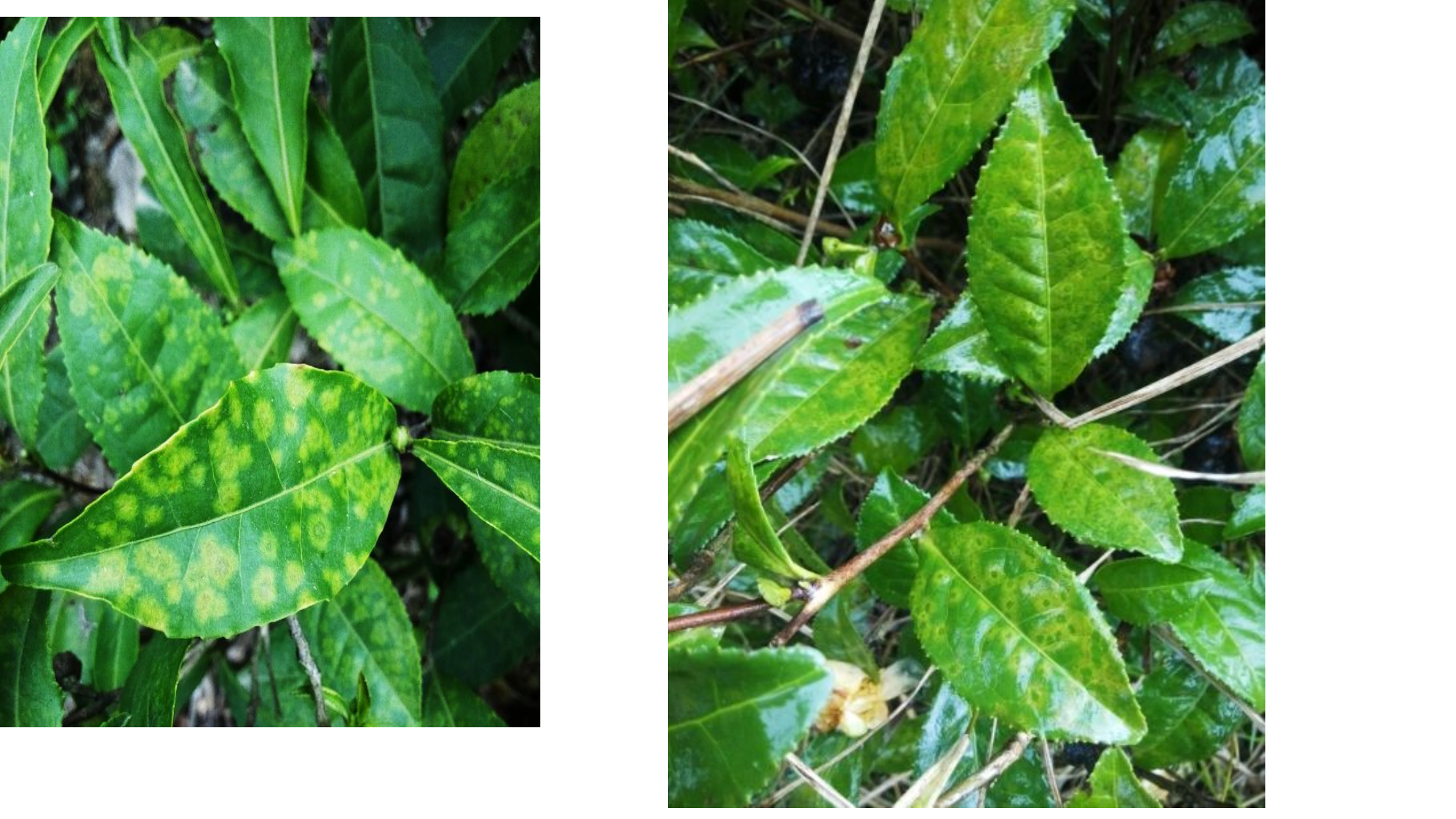

## Slide 3
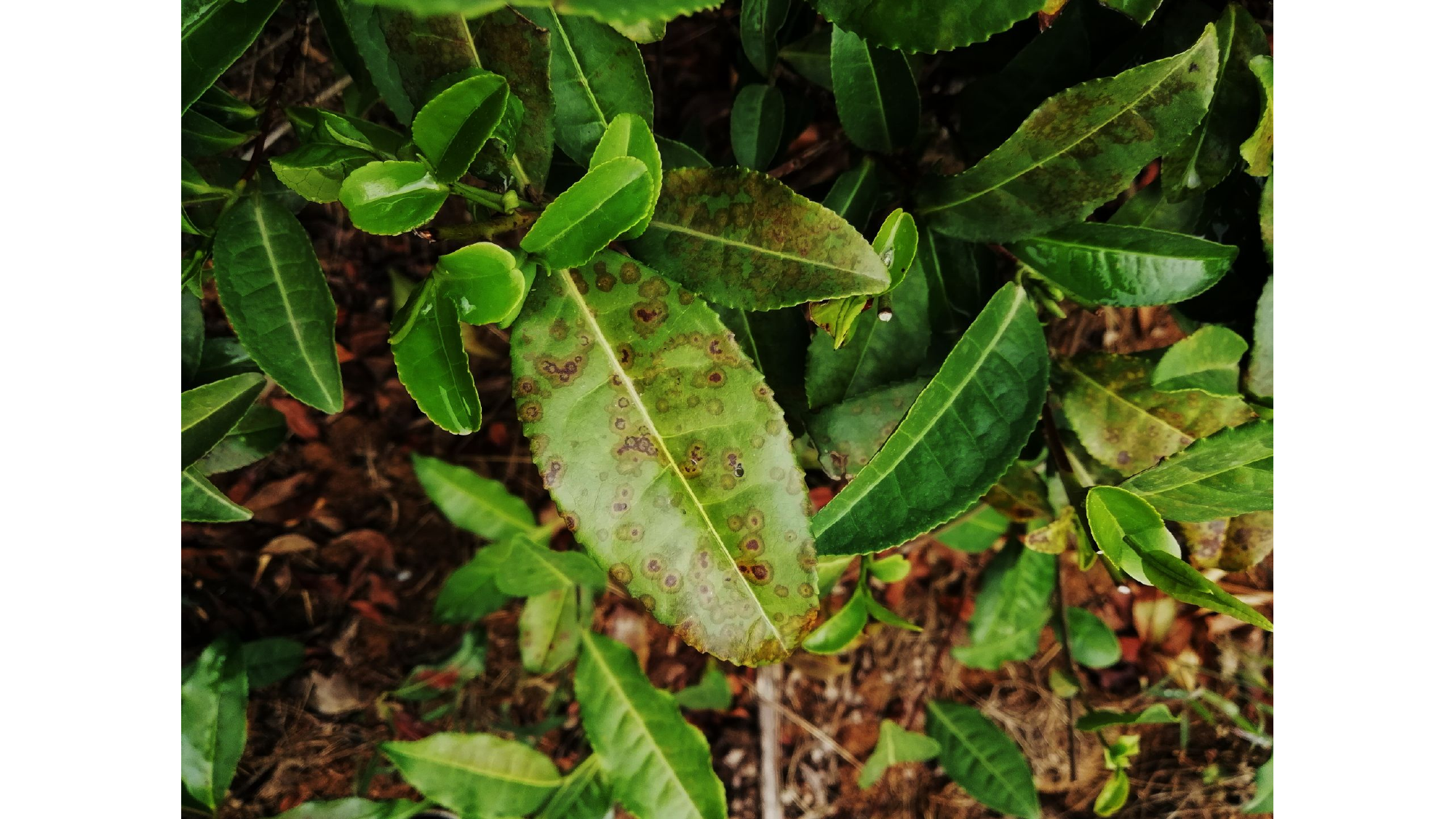

## Slide 4
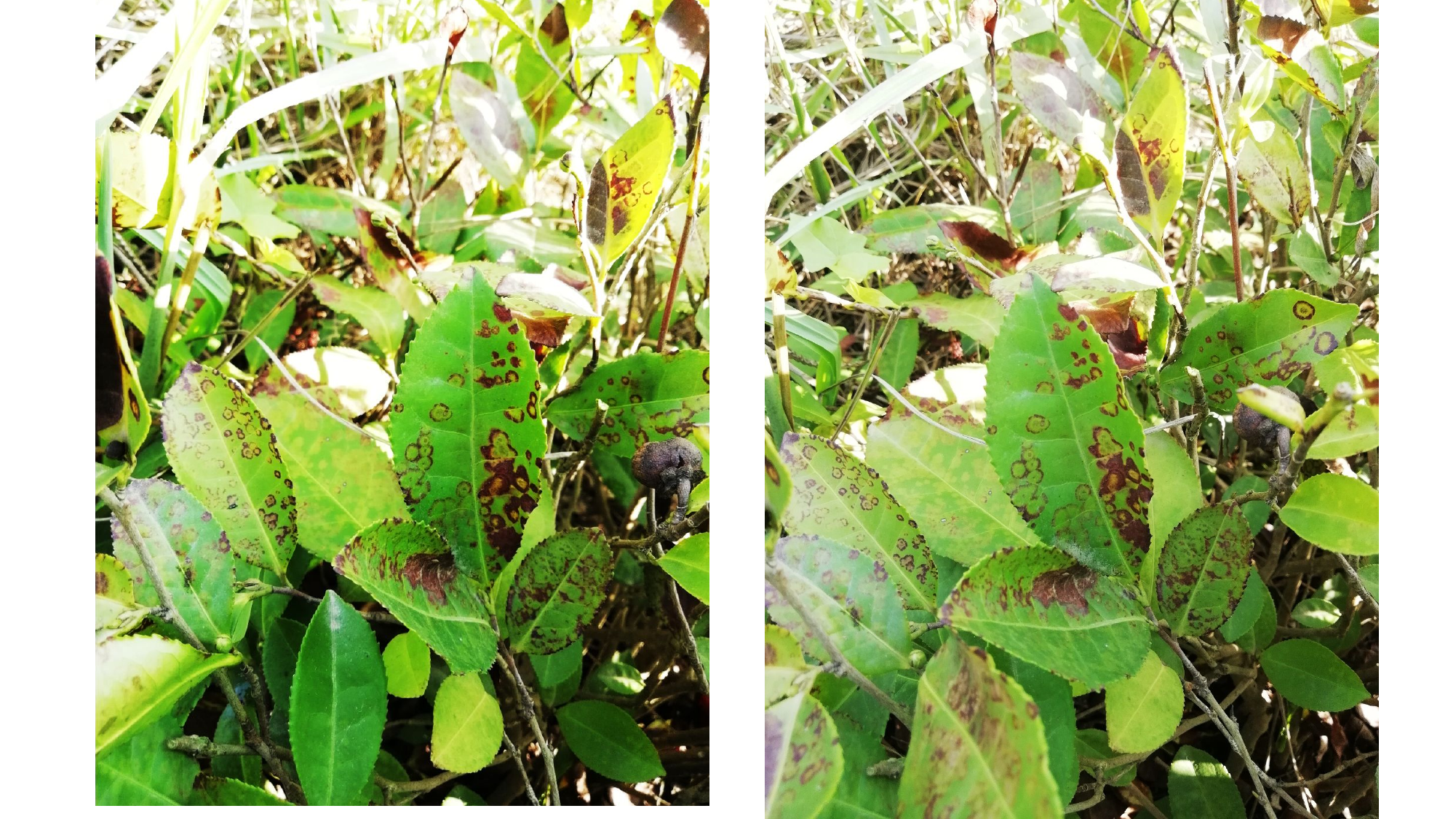

## Slide 5
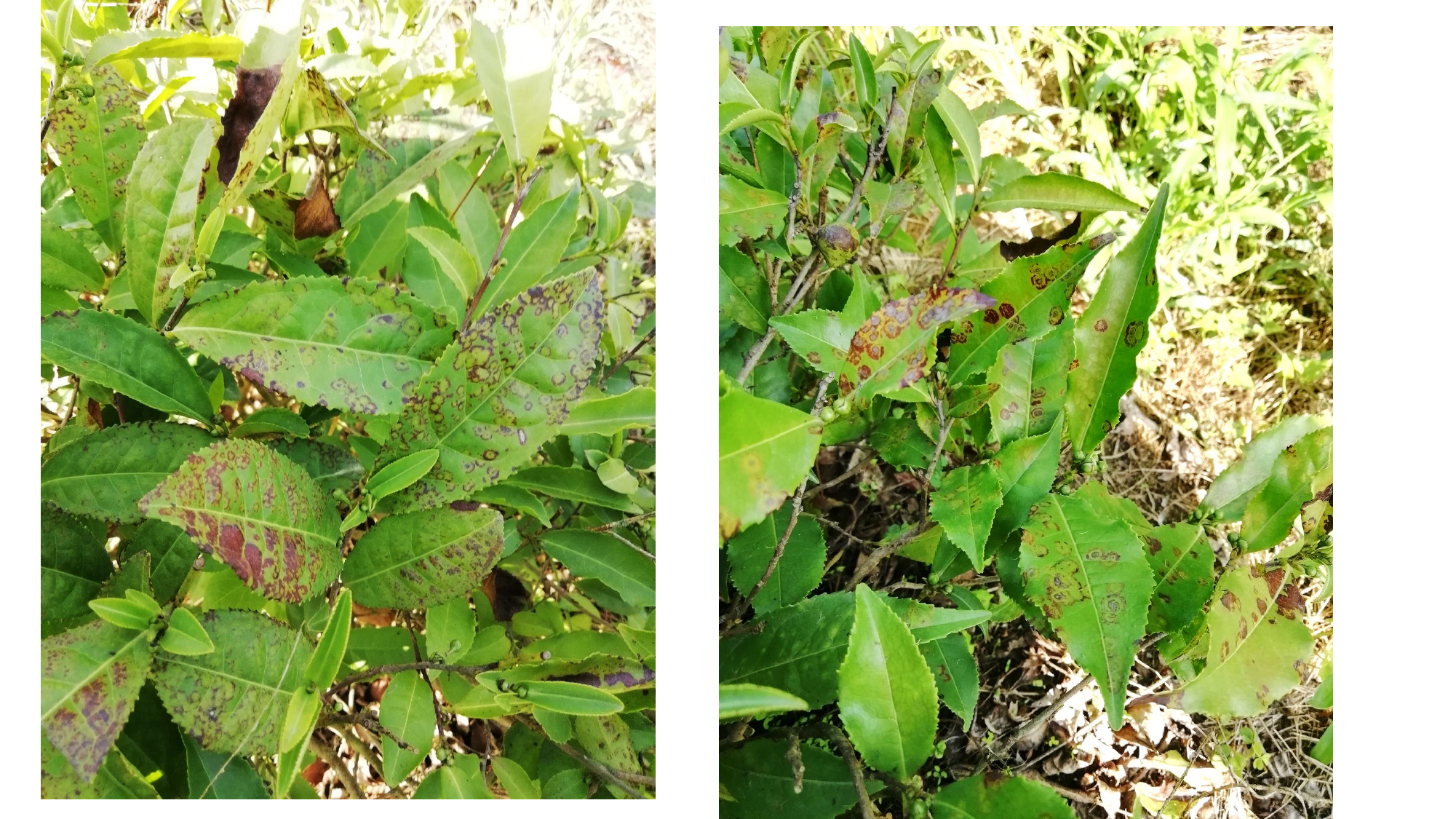

## Slide 6
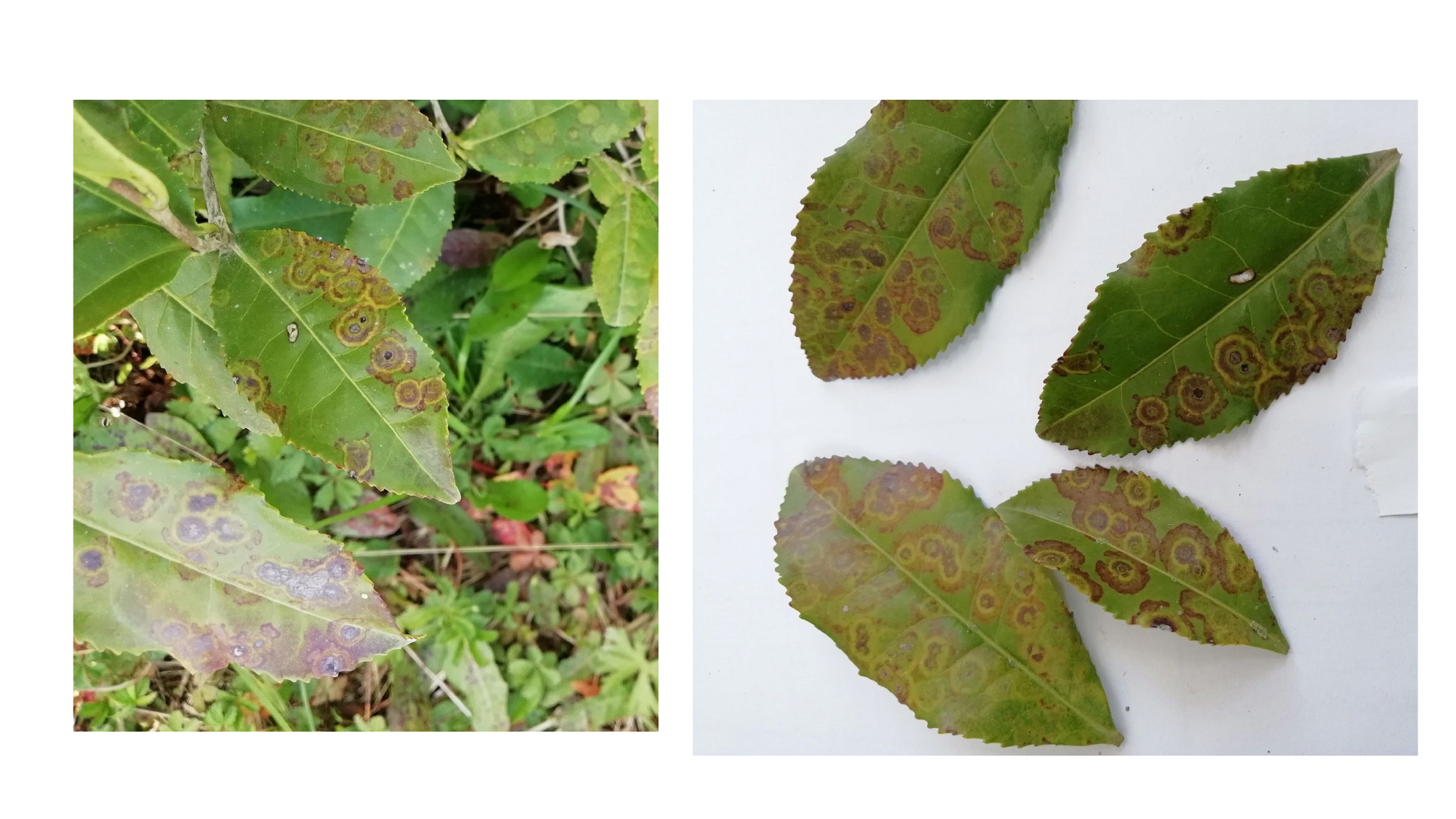

## Slide 7
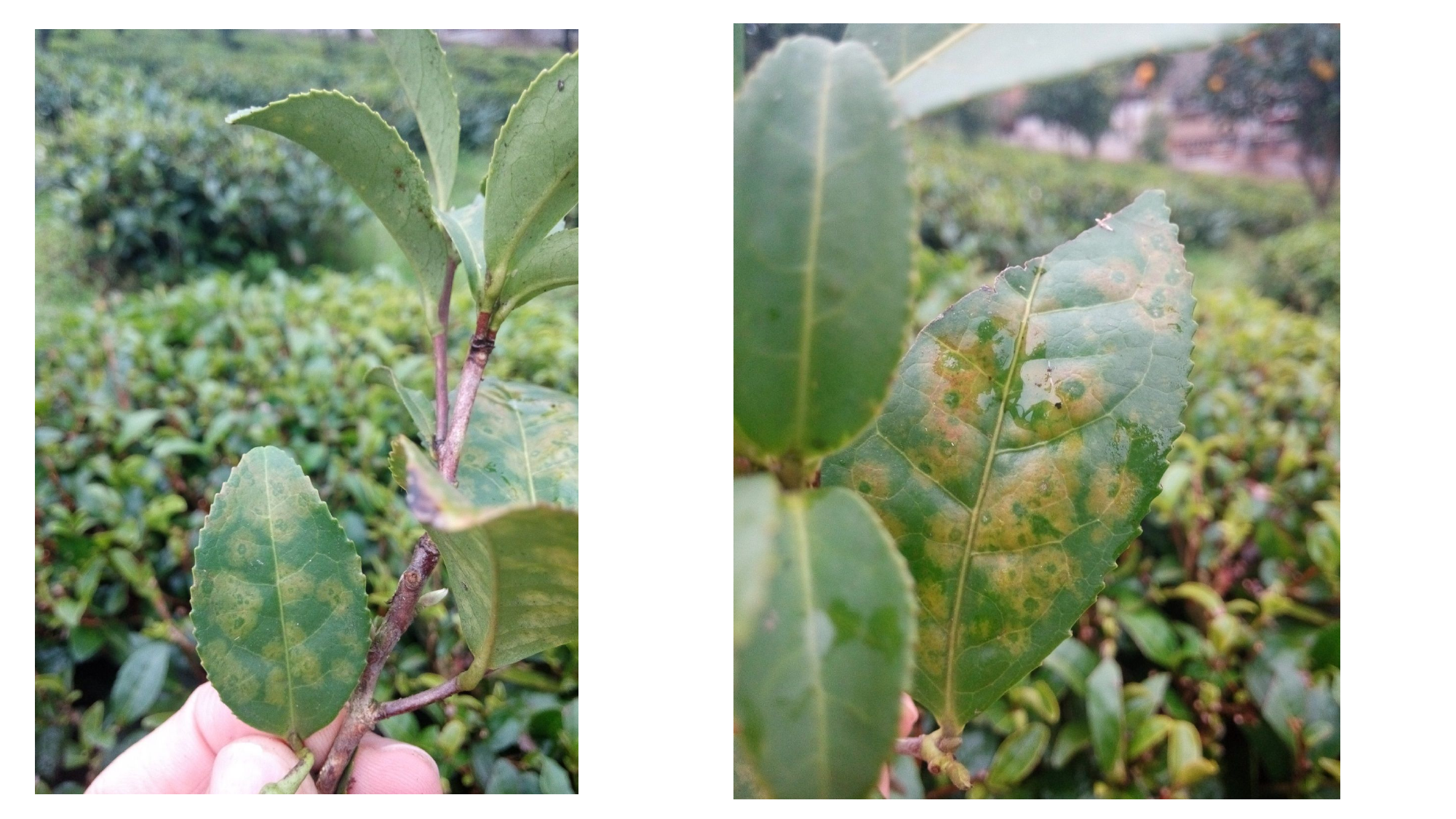

## Slide 8
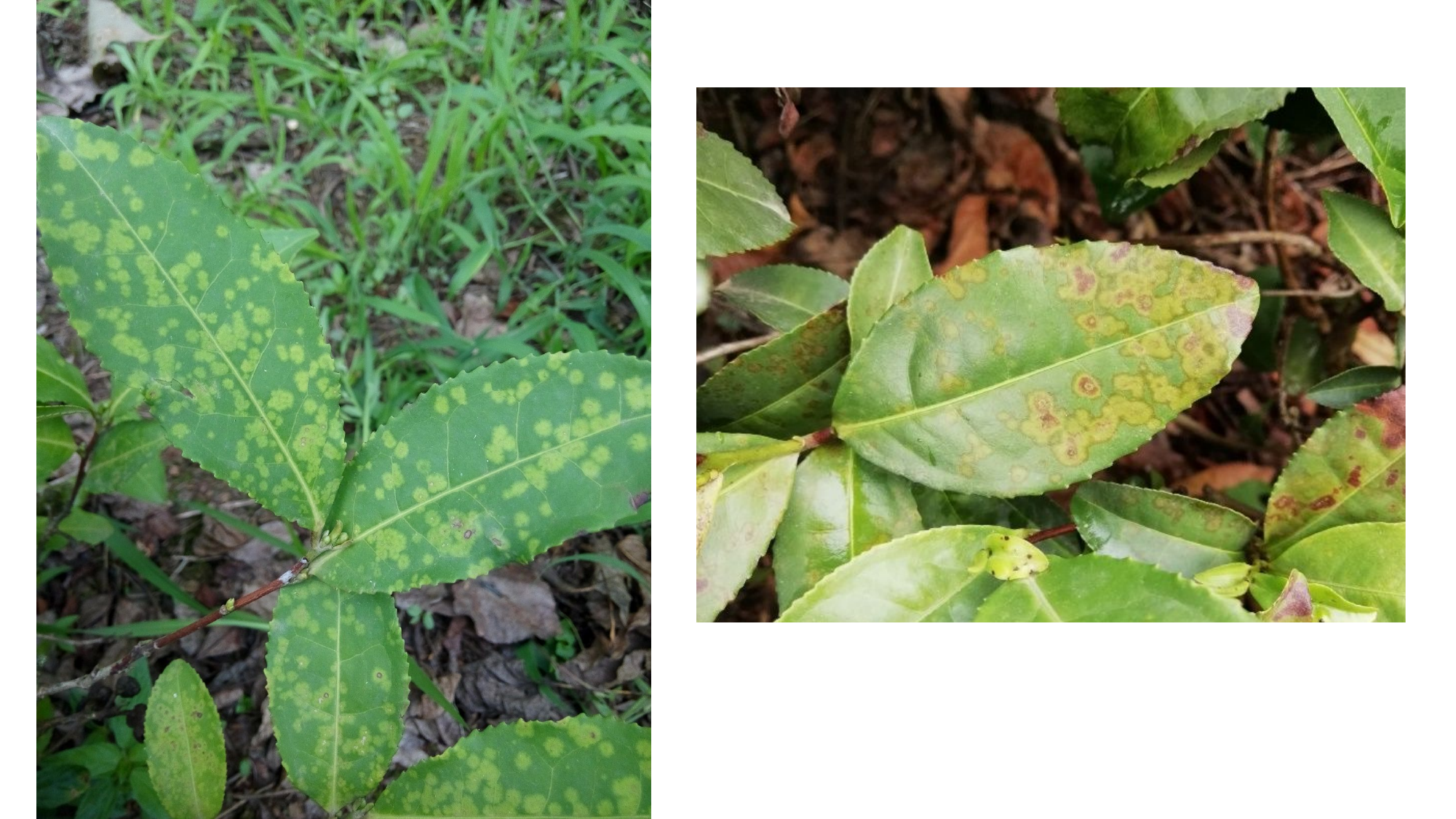

Supplement: Supplementary file 3 — Supplementary Information 3. [file 41598_2023_46654_MOESM3_ESM.pptx]
